# Determinants of variability in signature whistles of the Mediterranean common bottlenose dolphin

**DOI:** 10.1038/s41598-022-10920-7

**Published:** 2022-05-26

**Authors:** Gabriella La Manna, Nikolina Rako-Gospić, Daniela Silvia Pace, Silvia Bonizzoni, Lucia Di Iorio, Lauren Polimeno, Francesco Perretti, Fabio Ronchetti, Giancarlo Giacomini, Gianni Pavan, Giulia Pedrazzi, Helena Labach, Giulia Ceccherelli

**Affiliations:** 1 Dipartimento di Chimica e Farmacia, Università degli Studi di Sassari, Via Piandanna, 4, 07100 Sassari, Italy; 2 MareTerra Onlus - Environmental Research and Conservation, Regione Salondra 9, 07041 Alghero, Italy; 3 Blue World Institute of Marine Research and Conservation, Kaštel 24, 51551 Veli Lošinj, Croatia; 4 Department of Environmental Biology, Sapienza University of Rome, Piazzale Aldo Moro 5, 00185 Rome, Italy; 5 Dolphin Biology and Conservation, Via Cellina 5, 33084 Cordenons, Italy; 6 CNRS Centre de Formation et de Recherche sur les Environnements Méditerranéens, UMR 5110, Université de Perpignan Via Domitia, 52 avenue Paul Alduy, 66860 Perpignan, France; 7 Chorus Institute, 5 rue Gallice, 38100 Grenoble, France; 8 Jacksonville University, 2800 University Blvd North, Jacksonville, FL 32211 USA; 9 Department of Earth and Environmental Sciences, CIBRA, University of Pavia, Via Taramelli 24, 27100 Pavia, Italy; 10 MIRACETI, Place des Traceurs de Pierres, 13500 Martigues, France

**Keywords:** Behavioural ecology, Animal behaviour

## Abstract

One of the most studied aspects of animal communication is the acoustic repertoire difference between populations of the same species. While numerous studies have investigated the variability of bottlenose dolphin whistles between populations, very few studies have focused on the signature whistles alone and the factors underlying differentiation of signature whistles are still poorly understood. Here we describe the signature whistles produced by six distinct geographical units of the common bottlenose dolphin (*Tursiops truncatus*) in the Mediterranean Sea and identify the main determinants of their variability. Particularly, the influence of the region (proxy of genetic distance), the geographic site, and the environmental (sea bottom-related) and demographical (population-related) conditions on the acoustic structure of signature whistles was evaluated. The study provides the first evidence that the genetic structure, which distinguishes the eastern and western Mediterranean bottlenose dolphin populations has no strong influence on the acoustic structure of their signature whistles, and that the geographical isolation between populations only partially affected whistle variability. The environmental conditions of the areas where the whistles developed and the demographic characteristics of the belonging populations strongly influenced signature whistles, in accordance with the “acoustic adaptation hypothesis” and the theory of signature whistle determination mediated by learning.

## Introduction

Acoustic signals are widely used in animals, from insects to mammals, and can be involved in a variety of contexts such as sexual selection, social cohesion, individual and group recognition, resource defence and competition^1^. Cetaceans rely on acoustic communication for orientation, locating food, reproduction and avoiding predators^2^. Consequently, they have a varied and complex acoustic repertoire, used to transmit information related to the sender identity (individual, social group, population, species), sender position and behavioural contexts^3^. Some of the most studied cetacean species produce individual signals aimed to identify the emitter, as in *Tursiops* spp*.*^4,5^, or have acoustic repertoires able to identify family group or population, as in killer whales (*Orcinus orca*) and sperm whales (*Physeter macrocephalus*)^6–8^.

One of the most studied aspects of animal communication is the acoustic difference between closely related species or between populations of the same species^1,9^. Several processes have been proposed as determinants in the acoustic difference among populations of the same species: (i) the acoustic traits of the environment where the populations live mediate the acoustic signals’ evolution due to different sound propagation and ambient noise (the “acoustic adaptation hypothesis”^10^; (ii) social and cultural selection, especially when vocal learning is involved in the development of acoustic behaviour^1^; (iii) genetic causes, which may be further related to the geographic isolation between populations^11,12^.

The bottlenose dolphin, *Tursiops* spp., lives in fission–fusion societies^13^ where individual recognition, contact maintenance, and group coordination are mediated by frequency-modulated, narrow-band acoustic signals, called “whistles”^14,15^. Those whistles characterized by a stereotyped frequency modulation pattern (or contour) and used to identify the emitter are known as “signature whistles”^14^. They are primarily produced when an animal is separated by the conspecifics^4,5,23,65^ and to ensure social cohesion^14^. In fact, in captivity signature whistles are mainly produced when animals are isolated from the rest of the group^5,23,26^, while in wild dolphins, signature whistles account for 38–70% of the whole whistles’ repertoire^16–18^.

The signature whistle develops during the first year of a dolphin’s life and seems to be modelled hearing whistles from conspecifics^19–22^ and through vocal production learning^14,23^. Data from both captivity and the wild have partially excluded a strict genetic determination of signature whistle structure^19,22,24,28^.

The contour remains stable for decades^24^, even if a few situations are known to be responsible for its changes: (i) males can change their whistles contour in the attempt to resemble those of their alliance partners^25^; (ii) subtle changes (such as in the start frequency or bandwidth) may occur as consequence of motivational states. Thus, in addition to the information of emitter’s identity, the signature whistle may transmit context-related information^26^.

Since signature whistles are likely developed through vocal production learning^14^, as a young animal learns its vocalizations from its neighbours, a geographic variation between the signature whistles of different populations can be assumed. Attempts to characterise the signature whistle repertoire of bottlenose dolphin sub-populations have been done only in a few areas, such as Florida^17,27^, Scotland^28,29^, Namibia^30^, and Portugal^31^. In recent years the number of this kind of studies has increased thanks to development and application of the SIGnature IDentification method (SIGID^51^), based on the temporal patterning and stereotyped structure of the signature whistles^14,29,30,68^. While numerous studies have investigated the variability of the bottlenose dolphin whistles between populations^32–36^, with no distinction between signature and non-signature whistles, very few studies have focused on the signature whistles variability alone^30^. Thus, the factors underlying differentiation of signature whistles are still poorly understood and the investigation of signature whistles variability between populations may clarify the development process and evolution of this call type. The aim of this study was to describe the signature whistles produced by distinct geographical units of common bottlenose dolphin (*Tursiops truncatus*) in different Mediterranean sites and identify the determinants of their variability. Variability among populations may arise due to their relative geographic isolation and/or genetic distance. A genetic differentiation between the western and the eastern Mediterranean bottlenose dolphin populations exists^37^ and could influence signature whistles. Furthermore, when acoustic signal development is mediated by vocal learning, differences in social structure, population size, and connection between adjacent populations may affect acoustic variability. In the end, the environmental conditions related to water depth, substrate type and habitat, may influence acoustic signals through their effect on sound transmission^38^. Then, to disentangle the potential causes of variability and identify the main determinants, the acoustic structure of signature whistles (in terms of fundamental frequencies, frequency modulation and duration) was investigated and the influence of the Mediterranean region (as a proxy of genetic distance), the geographic site, and the environmental (sea bottom-related) and demographical (population-related) conditions were evaluated.

## Results

A total of 188 h of recordings were collected, from which we overall extracted 2036 good quality signature whistle contours (SWs). Particularly, 101 SWs from Port Cros (PC) (corresponding to 11 SW-IDs), 166 from Ostia-Fiumicino (FI) (corresponding to 17 SW-IDs), 925 from Alghero (AL) (corresponding to 58 SW-IDs), 406 from Lampedusa (LA) (corresponding to 37 SW-IDs), 83 from Gulf of Corinth (GC) (corresponding to 12 SW-IDs), and 346 from Cres-Lošinj (CL) (corresponding to 33 SW-IDs) (see Supplementary Table S1 and Fig. S1 online).

### Similarity between SWs

**Figure 1:**
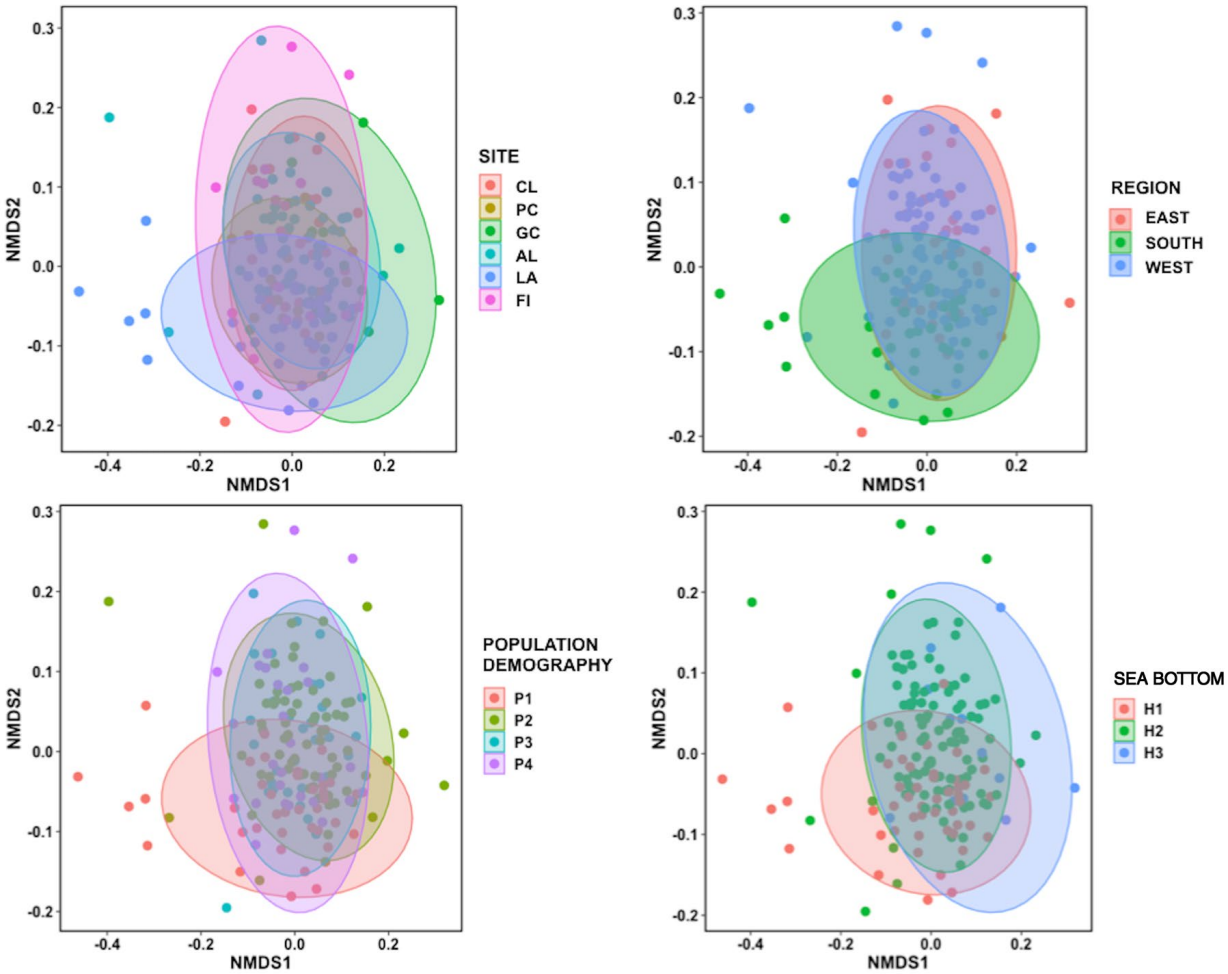
Multidimensional scaling plots showing the similarity of SWs grouped by site, region, population demography and sea bottom. PC (Port Cros); Al (Alghero); FI (Ostia-Fiumicino); LA (Lampedusa); GC (Gulf of Corinth); CL (Cres and Losjni).

SW structure was affected by all the factors considered as indicated by the nMDS (stress = 0.15; Fig. 1). This is confirmed by one-way non-parametric similarity analyses (Anosim) that identified a significant effect of region (*p* = 0.004, R = 0.081), site (*p* = 0.001, R = 0.14), sea bottom (*p* = 0.001, R = 0.15) and population demography (*p* = 0.032, R = 0.057).

### Collinearity among SW variables

**Figure 2:**
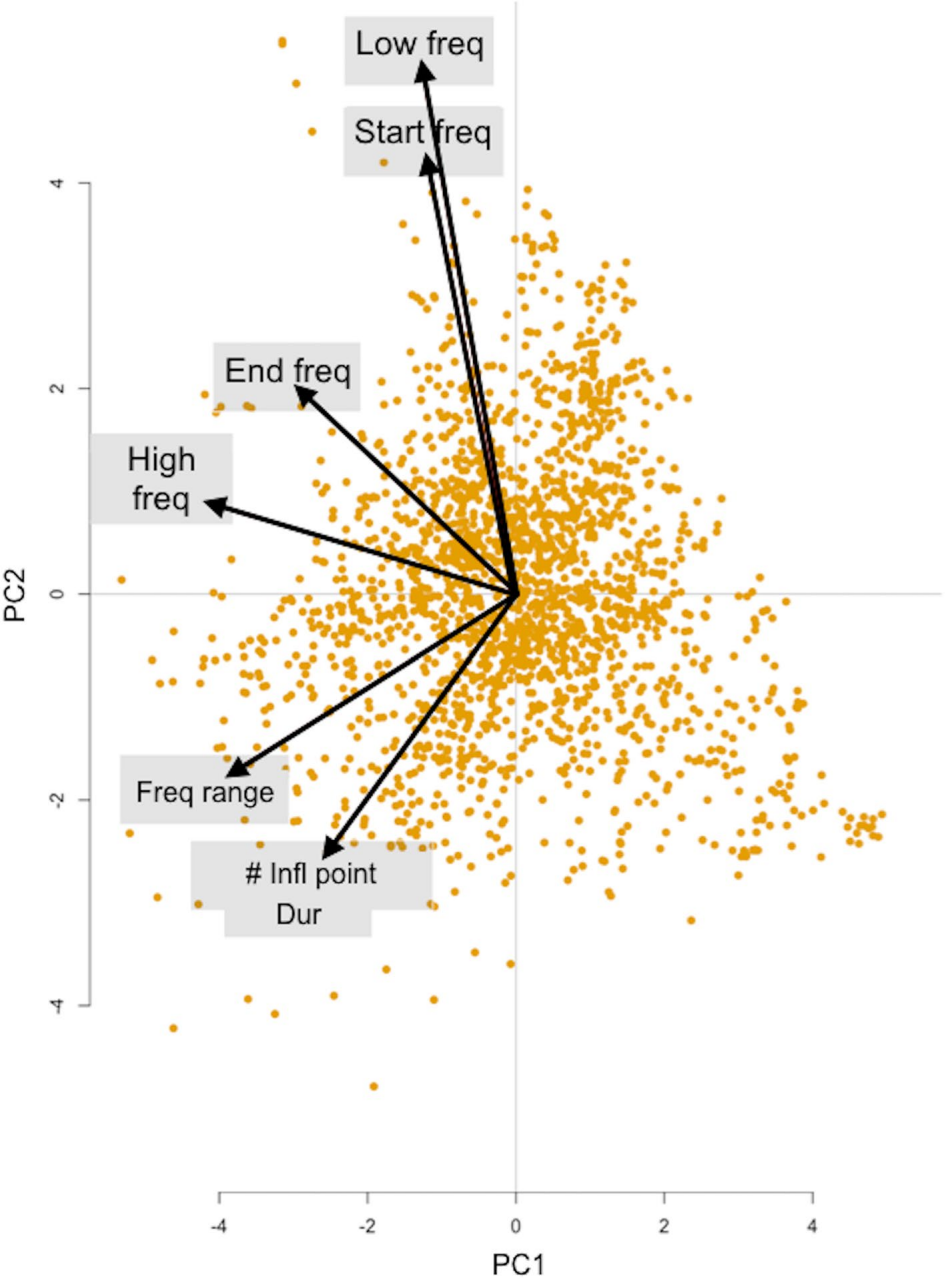
PCA biplot displays the information on correlation among variables. The directions of the arrows show the relative loadings of the parameters on PC1 and PC2.

The seven SW acoustic characteristics were highly collinear, thus a PCA was applied. The first principal component (PC1) was negatively correlated with max and end frequencies (and explained 36% of the variance); the second principal component (PC2) was positively correlated with min and start frequencies (and explained 26% of the variance); the third principal component (PC3) was positively correlated with duration and number of inflection points and negatively correlated with frequency range (and explained 21% of the variance) (Fig. 2).

### Influence of region, site, sea bottom and population demography

We used GLMMs on PC1, PC2 and PC3 to investigate the influence of region, site, sea bottom and population demography with separated models.

**Figure 3:**
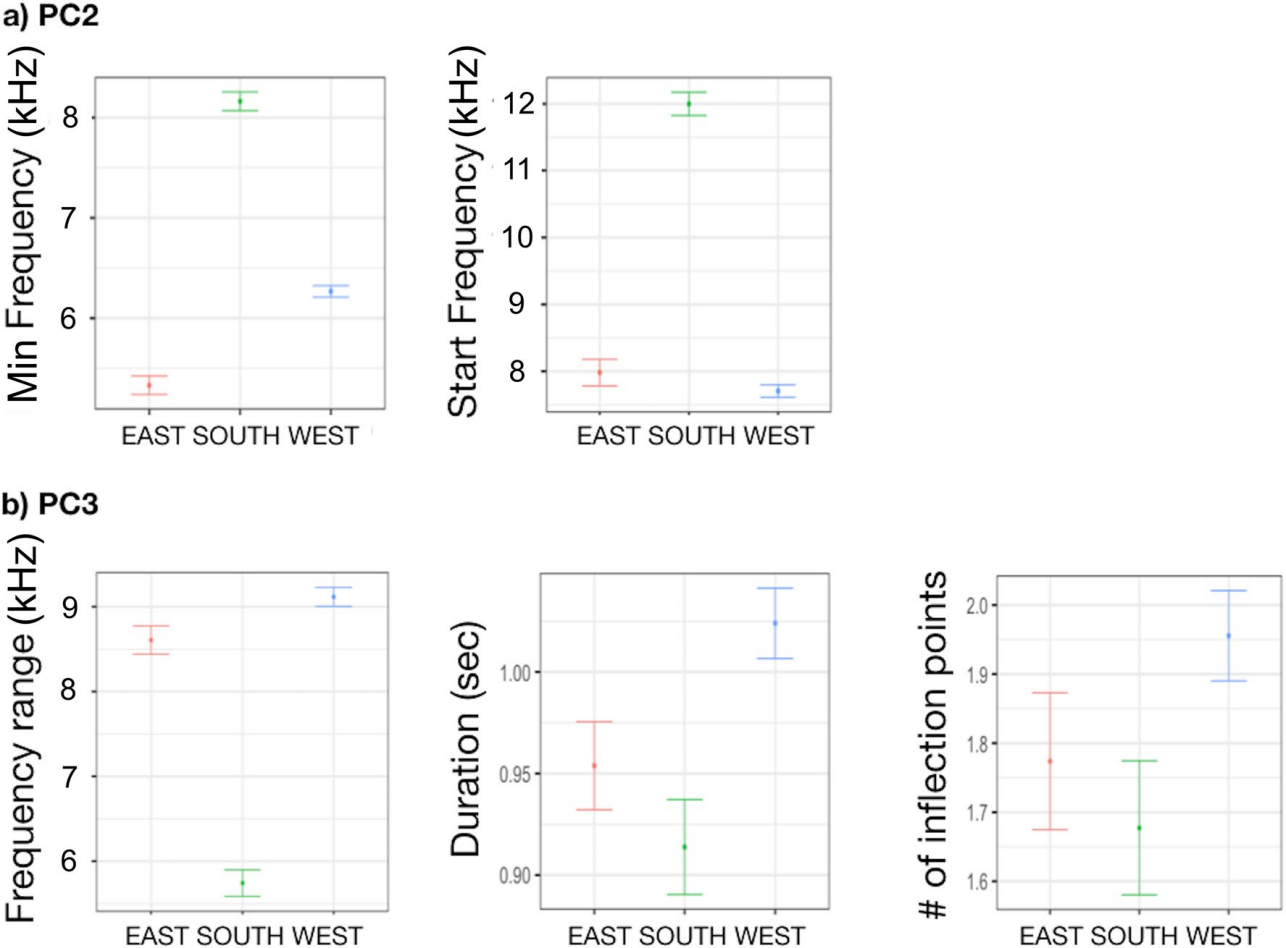
Effect of ‘region’ on (**a**) min and start frequencies (PC2) and (**b**) frequency range, duration and number of inflection points (PC3).

Only PC2 and PC3 were significantly associated with region (Table 1). PC2 and PC3 were both higher in the SOUTH compared to the EAST and WEST (Fig. 3). Particularly, min and start frequencies were higher in the SOUTH compared to WEST and EAST, while frequency range was lower. Duration and number of inflection points did not change (Table 1, Fig. 3).

**Table 1:** Generalized linear mixed-effect model (GLMM) with ‘region’ as explanatory variable on PC1, PC2 and PC3.

PC1					
Fixed effect	Value	SE	DF	t-value	*p*-value
Intercept	0.1536	0.2102	1860	0.7304	0.4652
SOUTH	0.2585	0.3122	164	0.8280	0.4089
WEST	− 0.3822	0.2596	164	− 1.4725	0.1428
***Random effects***	**SD (Intercept)**	**Residual**			
SW-ID (Intercept)	1.3760	0.7412			

PC2					
Fixed effect	Value	SE	DF	t-value	*p*-value
Intercept	**− **0.6924077	0.1679645	1860	**− **4.122344	** < 0.0001**
SOUTH	1.3577182	0.2492973	164	5.446181	** < 0.0001**
WEST	0.3802016	0.20728	164	1.834242	0.0684
***Random effects***	**SD (Intercept)**	**Residual**			
SW-ID (Intercept)	1.0907	0.6805			

PC3					
Fixed effect	Value	SE	DF	t-value	*p*-value
Intercept	0.0828627	0.1653987	1860	0.500988	0.6164
SOUTH	0.8489706	0.2456436	164	3.456107	**0.0007**
WEST	**− **0.2037748	0.2042081	164	**− **0.997878	0.3198
***Random effects***	**SD (Intercept)**	**Residual**			
SW-ID (Intercept)	1.081996	0.5892998			

The upper section shows the significant effects of the assessed explanatory variables. Value, standard errors (SE), t-values, and significance level (*p*-value) for variables retained in the model are provided for fixed effects (explanatory variables), while estimates of the standard deviations (SD) are reported for random effects (SW-ID). Significant level in bold.

**Figure 4:**
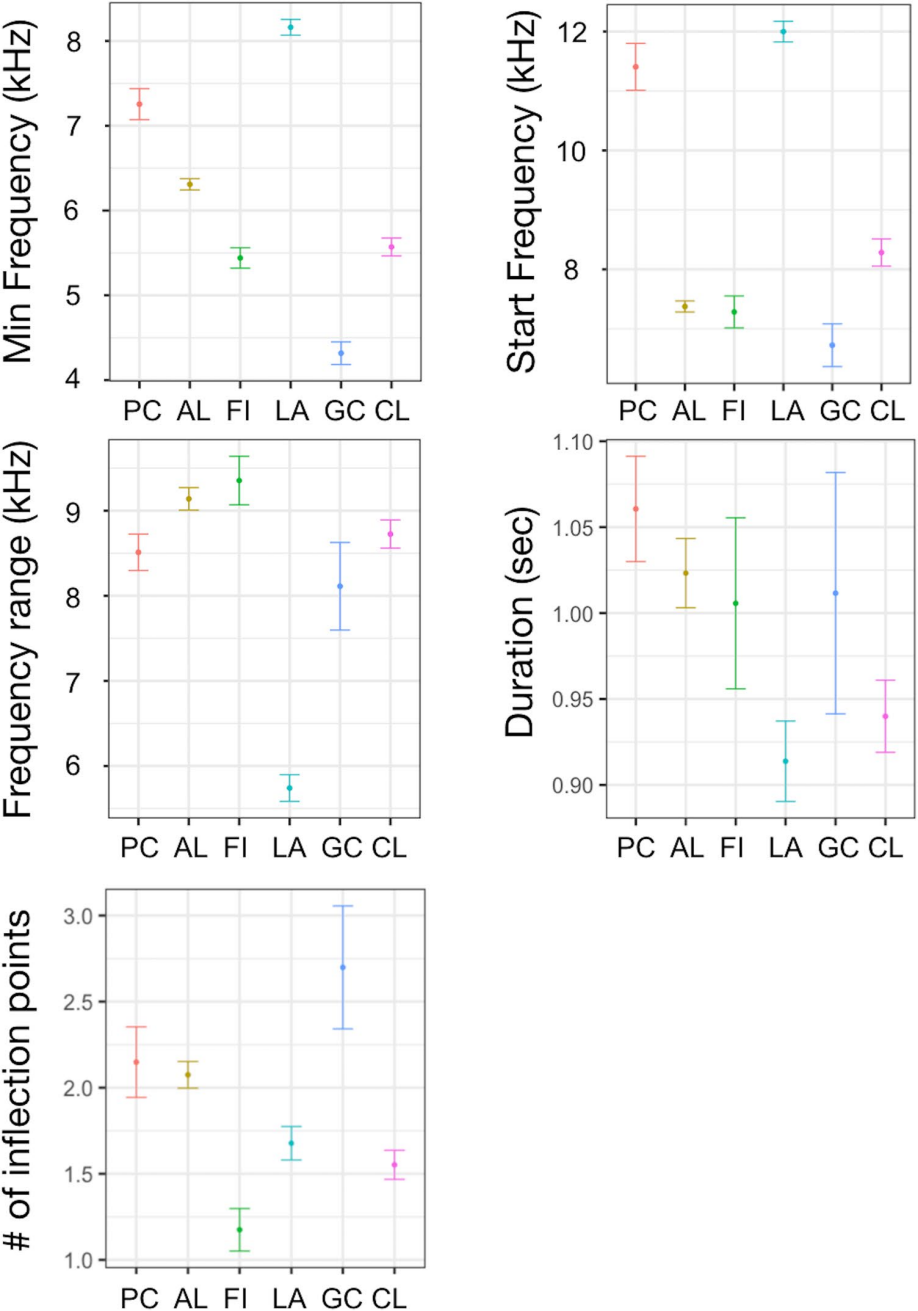
Effect of ‘site’ on (**a**) min and start frequencies (PC2) and (**b**) frequency range, duration and number of inflection points (PC3).

Only PC2 and PC3 were significantly associated with site, while no differences were found on PC1 (Table 2, Fig. 4). PC2 (min and start frequencies) were higher in PC and LA and lower in GC. PC3 was higher in LA, thus the negatively correlated frequency range was lower, while duration and number of inflection points did not change (Table 2, Fig. 4).

**Table 2:** Generalized linear mixed-effect model (GLMM) with ‘site’ as explanatory variable on PC1, PC2 and PC3.

PC1					
Fixed effect	Value	SE	DF	t-value	*p*-value
Intercept	0.0489	0.2463	1860	0.1985	0.8427
GC	0.3944	0.4782	161	0.8247	0.4108
PC	**− **0.4568	0.4925	161	**− **0.9276	0.3550
AL	**− **0.3268	0.3090	161	**− **1.0575	0.2919
LA	0.3632	0.3383	161	1.0738	0.2845
FI	0.0021	0.4211	161	0.0049	0.9961
***Random effects***	**SD (Intercept)**	**Residual**			
SW-ID (Intercept)	1.3821	0.7412			

PC2					
Fixed effect	Value	SE	DF	t-value	*p*-value
Intercept	**− **0.3675	0.1880	1860	**− **1.9547	**0.0508**
GC	**− **1.2239	0.3655	161	**− **3.3483	**0.0010**
PC	0.8201	0.3760	161	2.1812	**0.0306**
AL	**− **0.0692	0.2357	161	**− **0.2936	0.7695
LA	1.0342	0.2580	161	4.0076	**0.0001**
FI	**− **0.0139	0.3210	161	**− **0.0433	0.9655
***Random effects***	**SD (Intercept)**	**Residual**			
SW-ID (Intercept)	1.1044	0.6804			

PC3					
Fixed effect	Value	SE	DF	t-value	*p*-value
Intercept	**− **0.0810	0.1905	1860	**− **0.4254	0.6706
GC	0.6175	0.3700	161	1.6692	0.097
PC	0.5572	0.3809	161	1.4628	0.1455
AL	**− **0.0675	0.2390	161	**− **0.2823	0.7781
LA	1.0128	0.2616	161	3.8712	**0.0002**
FI	**− **0.3310	0.3256	161	**− **1.0164	0.3109
***Random effects***	**SD (Intercept)**	**Residual**			
SW-ID (Intercept)	1.0676	0.5893			

The upper section shows the significant effects of the assessed explanatory variables. Value, standard errors (SE), t-values, and significance level (*p*-value) for variables retained in the model are provided for fixed effects (explanatory variables), while estimates of the standard deviations (SD) are reported for random effects (SW-ID). Significant level in bold.

**Figure 5:**
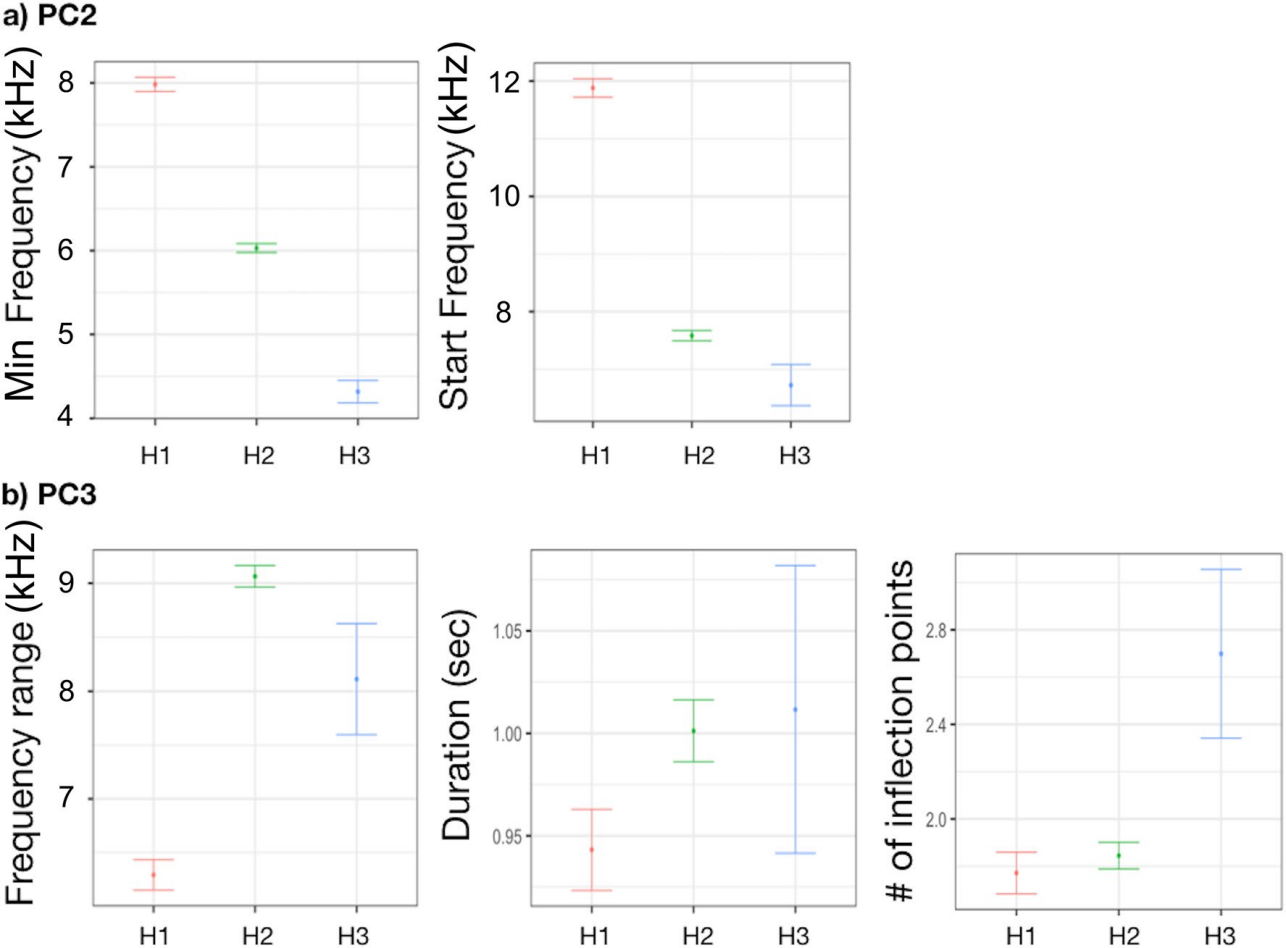
Effect of ‘sea bottom’ on (**a**) min and start frequencies (PC2) and (**b**) frequency range, duration and number of inflection points (PC3).

The cluster analysis grouped the sea bottom variables (habitat type and depth range) into three clusters: (i) H1 grouped together the sites (PC and LA) characterized by Posidonia bed, infralittoral sand and circalittoral coarse sediment bottoms not exceeding 150 m of depth; (ii) H2 grouped together the sites (AL, CL and FI) characterized by circalittoral coastal terrigenous muds and coarse sediment and muddy detritic bottoms not exceeding 100 m of depth; (iii) H3 corresponded with GC, characterized by open-sea detritic bottoms on shelf-edge, offshore circalittoral coastal terrigenous muds and bathyal mud, with a depth up to 500 m. Only PC2 and PC3 were significantly associated with sea bottom (Table 3). PC2 (min and start frequencies), were higher in H1 and lower in H3 (Fig. 5). PC3 was lower in H2 compared to H1 and H3 (Fig. 5). Particularly, frequency range was higher in H2 than in H1 and H3, while the number of inflection points was higher in H3.

**Table 3:** Generalized linear mixed-effect model (GLMM) with ‘sea-bottom’ as explanatory variable on PC1, PC2 and PC3.

PC1					
Fixed effect	Value	SE	DF	t-value	*p*-value
Intercept	0.2250	0.2045	1860	1.1005	0.2713
H2	**− **0.3503	0.2461	164	**− **1.4236	0.1565
H3	0.2182	0.4595	164	0.4748	0.6356
***Random effects***	**SD (Intercept)**	**Residual**			
SW-ID (Intercept)	1.3876	0.7412			

PC2					
Fixed effect	Value	SE	DF	t-value	*p*-value
Intercept	0.6181	0.1541	1860	4.0101	**0.0001**
H2	**− **1.0247	0.1855	164	**− **5.5244	**< 0.0001**
H3	**− **2.2093	0.3472	164	**− **6.3635	**< 0.0001**
***Random effects***	**SD (Intercept)**	**Residual**			
SW-ID (Intercept)	1.1489	0.6804			

PC3					
Fixed effect	Value	SE	DF	t-value	*p*-value
Intercept	0.8278	0.1573	1860	5.2639	**< 0.0001**
H2	**− **0.9977	0.1893	164	**− **5.2716	**< 0.0001**
H3	**− **0.2914	0.3535	164	**− **0.8243	0.4110
***Random effects***	**SD (Intercept)**	**Residual**			
SW-ID (Intercept)	1.0657	0.5893			

The upper section shows the significant effects of the assessed explanatory variables. Value, standard errors (SE), t-values, and significance level (*p*-value) for variables retained in the model are provided for fixed effects (explanatory variables), while estimates of the standard deviations (SD) are reported for random effects (SW-ID). Significant level in bold.

**Figure 6:**
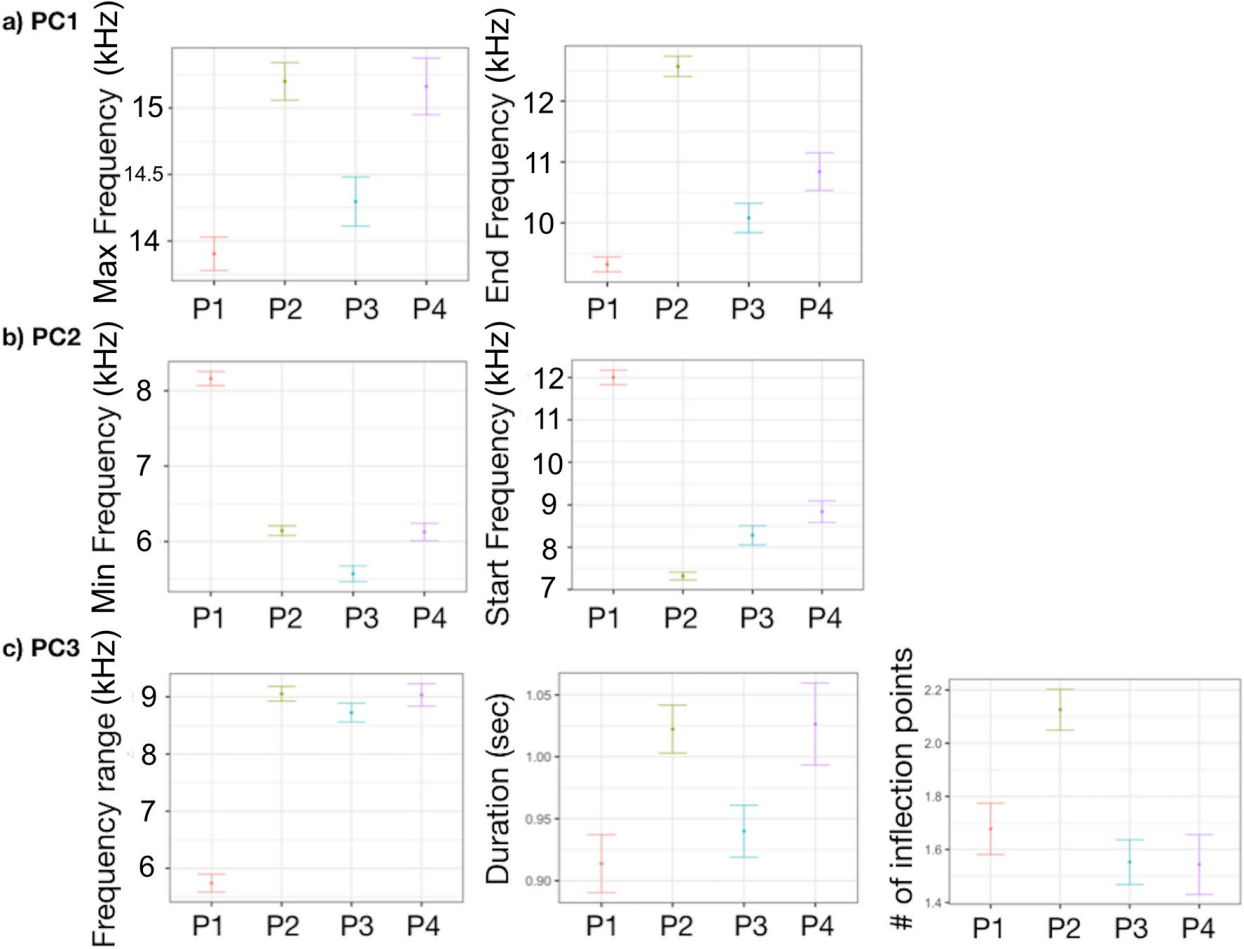
Effect of ‘population demography’ on (**a**) max and end frequencies (PC1), (**b**) min and start frequencies (PC2) and (c) frequency range, duration and number of inflection points (PC3).

Based on population demography, the cluster analysis grouped the six sites into four clusters: (i) P1 corresponded to LA, a quite large open population, with small group size and mostly transient individuals; (ii) P2 included AL and GC, the smallest sized populations with medium group size and mainly resident individuals; (iii) P3 corresponded to CL, an intermediate sized and isolated population, with large group size and mostly resident individuals; (iv) P4 included FI and PC, large sized open populations, with large group size and mostly transient individuals. All PCs were significantly associated with population demography (Table 4, Fig. 6). PC1 was higher in P1 respect to the others, thus max and end frequencies were both lower. PC2, thus min and start frequencies, were higher in P1. PC3 was higher in P1 and lower in P3. Particularly, frequency range and duration were lower in P1 while the number of inflection points was higher in P2 (Table 4, Fig. 6).

**Table 4:** Generalized linear mixed-effect model (GLMM) with ‘population demography’ as explanatory variable on PC1, PC2 and PC3.

PC1					
Fixed effect	Value	SE	DF	t-value	*p*-value
Intercept	0.4120742	0.2327578	1860	1.770399	0.0768
P2	**− **0.5661855	0.2885451	163	**− **1.962208	**0.0514**
P3	**− **0.363238	0.3395787	163	**− **1.069673	0.2863
P4	**− **0.5404204	0.3546808	163	**− **1.523681	0.1295
***Random effects***	**SD (Intercept)**	**Residual**			
SW-ID (Intercept)	1.3878	0.7412			

PC2					
Fixed effect	Value	SE	DF	t-value	*p*-value
Intercept	0.6653913	0.1837333	1860	3.621507	**0.0003**
P2	**− **1.3005563	0.227777	163	**− **5.70978	**< 0.0001**
P3	**− **1.0336319	0.2681912	163	**− **3.854086	**0.0002**
P4	**− **0.7211849	0.2799941	163	**− **2.575715	**0.0109**
***Random effects***	**SD (Intercept)**	**Residual**			
SW-ID (Intercept)	1.1046	0.6805			

PC3					
Fixed effect	Value	SE	DF	t-value	*p*-value
Intercept	0.9319	0.1827	1860	5.0997	**< 0.0001**
P2	**− **0.9624	0.2265	163	**− **4.2483	**< 0.0001**
P3	**− **1.0129	0.2666	163	**− **3.7991	**< 0.0001**
P4	**− **0.9970	0.2785	163	**− **3.5807	**< 0.0001**
***Random effects***	**SD (Intercept)**	**Residual**			
SW-ID (Intercept)	1.0812	0.5893			

The upper section shows the significant effects of the assessed explanatory variables. Value, standard errors (SE), t-values, and significance level (*p*-value) for variables retained in the model are provided for fixed effects (explanatory variables), while estimates of the standard deviations (SD) are reported for random effects (SW-ID). Significant level in bold.

## Discussion

SWs from the six studied populations were homogeneous within but distinct between sites, and the dissimilarity was most evident between the whistles from LA and those from the other sites. This pattern is also evident when the similarity is grouped by region: SWs from the south Mediterranean appeared more dissimilar than those from the west and the east, which instead overlapped. If the SWs’ dissimilarity was mainly related to the geographic isolation between individuals from the six sites and/or the genetic distance between eastern and western populations of the basin, a greater difference in PCs would have been observed. Instead, the largest difference concerns the SWs coming from LA in the South, with the only exception of PC2, and the related min and start frequencies which were lower in CL and GC in the East compared to PC and AL in the West. The highest difference found in the SWs from LA is consistent with the closest acoustic structure between whistles from LA and those from the Atlantic Ocean, compared to those from the western Mediterranean populations, found in a previous study^35^. Given the geographical position in the middle of the Strait of Sicily, a most frequent or recent contact between individuals from LA with those of the neighbouring populations that inhabit the waters of the Atlantic^35^ could explain the difference in SWs between the South and the other regions of the Mediterranean Sea. The case in which SWs dissimilarity was not related to a greater genetic distance between populations was already described by Gridley^28^, who found a similar pattern in the SWs from different African *Tursiops* spp. populations. Although it is known that the development of SWs does not have a strict genetic determination, but rather a determination mediated by learning^19,24^, the definitive understanding of how the genetic distance and the isolation between populations affect the acoustic variability of SWs is still unknown. However, our study provides the first evidence that the genetic structure which distinguishes the eastern and western Mediterranean bottlenose dolphin populations has no strong influence on the acoustic structure of their SWs. Furthermore, even the geographical isolation between populations of the investigated sites only partially influenced the SWs variability. Stronger evidence was obtained by associating the SWs to the type of sea bottom in which they developed and the demographic characteristics of the belonging populations.

The highest dissimilarity was found between SWs from H1 and H3. In the environment characterized by the presence of Posidonia bed (H1, corresponding to LA and PC), the SWs had the highest start and min frequencies and were shorter and less modulated compared to the SWs from H2 and H3. Since signature whistles are cohesion call, which are used to maintain group cohesion and facilitate mother-calf reunion, lower frequency and less modulated whistles could be preferred since they transmit further in the marine environment^57^. However, Quintana-Rizzo and Mann^58^ found that min frequency whistle attenuated up to seven times more in seagrass areas than in areas with other bottom type (mud or sandy-mud). Coherently, in H3 (GC), characterized prevalently by muddy and detritic bottom, min and start frequency were the lowest recorded and duration and number of inflection points were the highest. Few studies have been conducted to understand the role of depth in dolphin whistles, however, Buckstaff^16^ found lower minimum frequencies in deeper habitat and Gridley^28^ found longer whistles related to higher depth. These findings are coherent with the SWs characteristics of GC. Unfortunately, no other conclusion can be derived for the other sites, since they all have similar depth condition, thus this aspect needs further investigation.

The strongest influence on the variability of SWs was related to the population demography. The SWs of P1 (LA) and P4 (FI and PC) were the most dissimilar. These populations have the largest size and are composed of mostly transient individuals. A high number of sounds from conspecifics in large open populations can lead to the development of widely distinctive SWs to enhance recognition^57^. In P1, SWs had quite distinct acoustic characteristics: the lowest max and end frequencies, frequency range, duration, and number of inflection points. These characteristics are likely influenced by the combined effect of several factors (region and bottom type), rather than by the type of population alone, and this makes the interpretation of the results more complex.

In P2 (corresponding to AL and GC, the smallest sized populations), SWs had the highest number of inflection points. Further, GC had the highest variability in duration. In small populations, where the probability to meet the same individuals is high, different SWs duration and higher number of inflection points can enhance identity coding^59,60^, even if this pattern was found elsewhere, but in a larger population^28^.

Some methodological limitations need to be considered when interpreting the results of this study. Firstly, the sample size used for the analysis should be taken into consideration, since it may be not fully representative of the variability of SWs, especially in some sites. In fact, even if the 13 SW-IDs recorded in GC can be considered sufficiently representative of the SW repertoire of this population (composed of 38 individuals on average), the SW-IDs collected in PC and FI correspond to less than 5% of individuals in these large-sized populations.

Further, a limited number of the potential factors associated with the acoustic environment and whistle variability were considered in this study. For example, data on ambient noise and vessel traffic were not available for all sites, thus these factors could not be included. High noise levels caused by vessels can have a strong influence on whistle structure^36,61,62^ due to the need of making the signal more efficient in terms of transmission in noisy environments and to contrasting masking phenomena. However, a recent study^36^ compared the effect of noise on the whistles (both signature and variant) of two populations considered in the present study (AL and CL), showing different acoustic response to the increase of Sound Pressure Levels (in the 125, 500 and 1000 Hz octave bands) and boat presence between the two sites. This finding suggests that ambient noise levels alone are not sufficient to explain the variation in whistle acoustic structure. In fact, all the factors influencing local sound propagation together with the characteristics of boat traffic (in terms of quantity, type and size of boats) and the interaction of these factors with the behavior of dolphin groups and physiology of individuals should be considered. Here, only general, broad-scale environmental characteristics were used. Sound transmission in shallow water is highly variable and depends on bottom sediments, depth and slope^38^, but also on tidal events, temperature gradients, freshwater inputs, obstacles in the sound path^58^ and the interactive effect between the sediment and plants (such as seagrass meadows) and/or animals (like benthic in-fauna) that live on the bottom^63^. In the end, SW convergence between alliance members can reduce diversity in whistle types^25,64^. In the present study no data were available about the sex of the SW emitters neither on the association patterns between individuals. Thus, a most accurate description of the acoustic environment, the identity of the SW emitters and the social relationship between them should deserve attention in future studies to further understand their effect on the development of SWs.

## Methods

### Study areas

**Figure 7:**
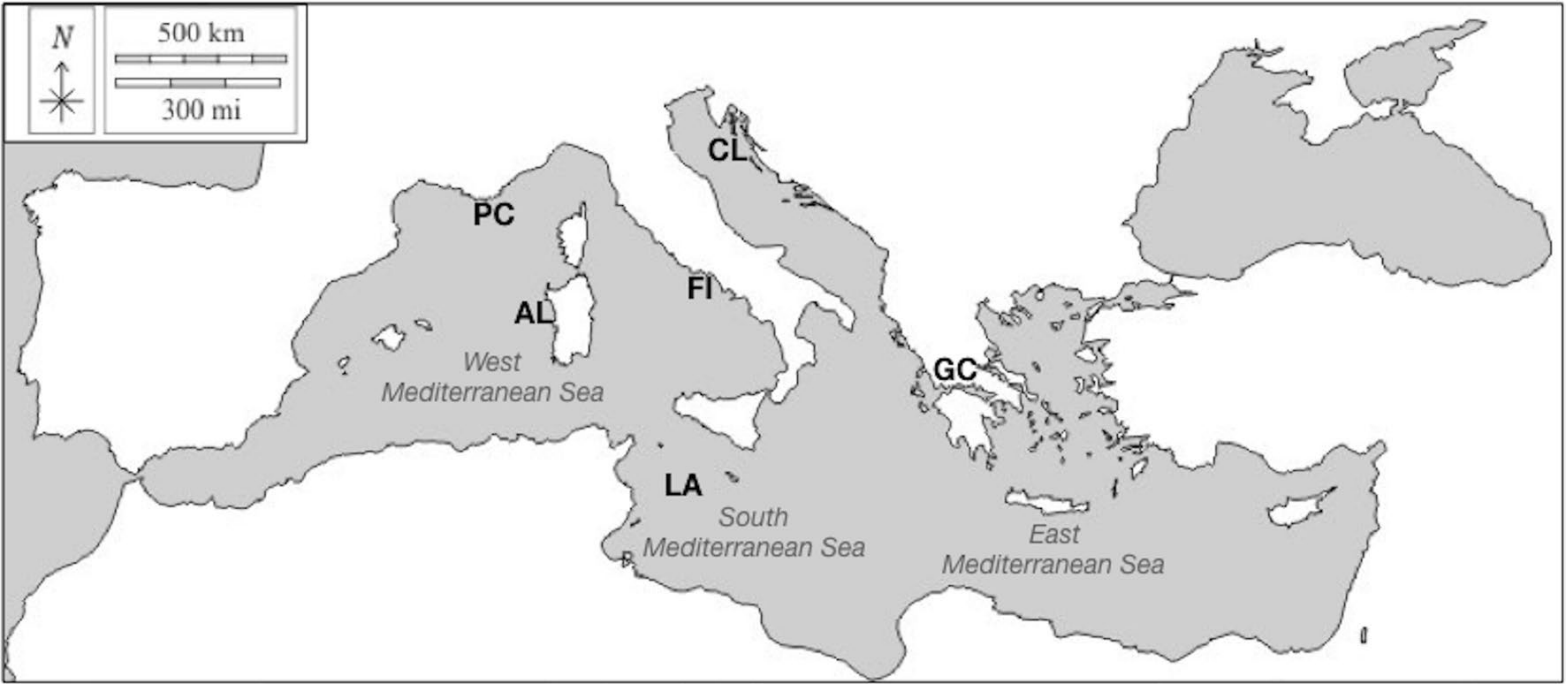
Map of the six study sites in the Mediterranean Sea. PC (Port Cros); Al (Alghero); FI (Ostia-Fiumicino); LA (Lampedusa); GC (Gulf of Corinth); CL (Cres and Losjni). The original map was downloaded from the free source https://d-maps.com/ and modified by Preview in Mac Os.

Based on physiographic, oceanographic, and biogeographic conditions, the Mediterranean Sea is divided into west and east basins, connected by the Strait of Sicily. To characterise the SWs of Mediterranean bottlenose dolphins we analysed acoustic data recorded in six different sites: Port Cros (French Riviera), Alghero (Sardinian Sea), and Ostia-Fiumicino (Tyrrhenian Sea), for the western basin, Cres-Lošinj (Adriatic Sea) and Gulf of Corinth (Ionian Sea), for the eastern basin, and Lampedusa (Strait of Sicily) in the southern Mediterranean Sea (Fig. 7).

### Port Cros (PC)

The acoustic recordings were collected in an area of approximately 13 km^2^ in the French Riviera, characterized by three main habitat types: Posidonia bed, circalittoral coarse sediment and infralittoral sand, with a bottom depth not exceeding 150 m. The bottlenose dolphin population inhabiting these waters has an estimated size of 223 individuals (95% CI = 152–385)^66,67^. The population is connected to adjacent populations of the Gulf of Lion and is mainly composed of transient individuals^66,67^. The mean group size is 16 individuals, with a range between 1 and 55^66,67^ (Table 5).

**Table 5:** Characteristics of the six populations studied for the four factors considered.

Region	Site	Sea bottom			Population demography				
		Cluster	Prevalent habitat type	Depth category	Cluster	Mean pop. size (95% CI)	Connection with other pop	Residency pattern	Mean group size (range)
WEST	PC	H1	Mediterranean circalittoral coarse sediment, Posidonia bed, Mediterranean Infralittoral sand	Shallow	P4	223 (152–385)	Yes	Mostly transient	16 (1–55)
WEST	AL	H2	Mediterranean circalittoral coarse sediment; Posidonia bed; Biocenosis of Mediterranean muddy detritic bottoms	Shallow	P2	76 (61–118)	Yes	Mostly resident	7 (1–17)
WEST	FI	H2	Biocenosis of Mediterranean offshore circalittoral coastal terrigenous muds; Biocenosis of Mediterranean muddy detritic bottoms; Mediterranean circalittoral coarse sediment	Shallow	P4	529 (456–614)	Yes	Mostly transient	15 (1–65)
SOUTH	LA	H1	Posidonia bed; Mediterranean Infralittoral sand; Mediterranean circalittoral coarse sediment	Shallow	P1	249 (162–449)	No	Mostly transient	4 (1–20)
EAST	CL	H2	Mediterranean infralittoral mud; Biocenosis of Mediterranean circalittoral coastal terrigenous muds; Mediterranean circalittoral coarse sediment	Shallow	P3	184 (152–250)	No	Mostly resident	22 (2–46)
EAST	GC	H3	Biocenosis of Mediterranean open-sea detritic bottoms on shelf-edge; Biocenosis of Mediterranean offshore circalittoral coastal terrigenous muds; Mediterranean bathyal mud	Depth	P2	38 (32–46)	Yes	Mostly resident	8 (1–28)

Region: (West, East, South). Site: Port Cros (PC), Alghero (AL), Ostia-Fiumicino (FI), Lampedusa (LA), Gulf of Corinth (GC), Cres and Losinj (CL). Depth category: i) shallow (< 120 m); depth (> 120 m) Sea bottom cluster: H1, H2, H3. Population demography: P1, P2, P3, P4. Prevalent habitat types and depth were retrieved from EMODnet platform (European Marine Observation and Data Network; https://emodnet.ec.europa.eu/en).

### Alghero (AL)

The acoustic recordings were collected in an area of about 450 km^2^ that includes Posidonia bed, circalittoral coarse sediment, muddy detritic bottoms, with a bottom depth up to 115 m. The bottlenose dolphin population inhabiting these waters has an estimated size of 76 individuals (95% CI = 61–118)^39^. Among the 122 photo-identified dolphins, at least 50% of them show a high level of site fidelity^40^ and they were sighted repeatedly every year and in different seasons. Nevertheless, the population seems neither closed nor isolated^40,41^. The mean size of the recorded groups is 7 individuals, with a range between 1 and 17 (Table 5).

### Ostia-Fiumicino (FI)

The acoustic recordings were collected in an area of approximately 1300 km^2^ of the Tyrrhenian Sea, characterized by three main habitat types: circalittoral coastal terrigenous muds, muddy detritic bottoms and circalittoral coarse sediment, with a bottom depth up to 100 m^42^. Here, three distinct groups were distinguished (resident, part-time, and transient), based on progressively lower degrees of site fidelity^43^. The part time and transient individuals accounts for most of the photo-identified individuals (78%). The estimated population size ranged between 77 (95% CI = 65–91) for the resident dolphins to 354 (95% CI = 288–312) for the transient ones, leading to a total of 529 individuals (95% CI = 456–614) for the whole population^43^. The mean group size is 15 individuals, with a range between 1 and 65^43^ (Table 5).

### Lampedusa Island (LA)

The acoustic recordings were collected in a 48 km^2^ area around Lampedusa Island located on the northern African continental shelf of the Strait of Sicily. The area includes Posidonia bed, infralittoral sand and circalittoral coarse sediment, with a bottom depth not exceeding 110 m. The estimated bottlenose dolphin population is 249 individuals (CI = 162–449)^44^, but likely these dolphins are part of a larger population^44^. The mean group size is 4, with a range between 1 and 20^45^ (Table 5).

### Gulf of Corinth (GC)

The acoustic recordings were collected along the northern coast of the Gulf where waters are characterized by open-sea detritic bottoms on shelf-edge, offshore circalittoral coastal terrigenous muds and bathyal mud, and bottom depth of 500 m (although the maximum depth considering the entire Gulf is 900 m). Here, a population of 38 individuals (95% CI = 32–46) was estimated^46^. Most individuals show high resighting frequency, but some dolphins are known to move in and out from the Gulf^47,48^. The mean group size is 8, with a range between 1 and 28^48^ (Table 5).

### Cres and Lošinj (CL)

The acoustic recordings were collected in an area of about 2000 km^2^ in the north-eastern Adriatic Sea. These waters are characterized by numerous uninhabited small islands and islets, infralittoral mud, circalittoral coastal terrigenous muds and circalittoral coarse sediment, with an average bottom depth of 70 m. Here, the population size was estimated to 184 individuals (95% CI = 152–250)^49^. The high sighting frequency of known individuals indicate their long-term fidelity to the region^50^. The mean size of the recorded groups is 22, with a range between 2 and 46^36^ (Table 5).

### Acoustic data collection

Acoustic recordings were collected with different methods and equipment, in different years and by different research groups (see Table 6). When the recordings were obtained by means of PAM (Passive Acoustic Monitoring) devices deployed on the sea bottom, as in LA, species identification was not visually confirmed. Nevertheless, the depths (< 40 m) and distances from the coast (< 1.5 km) were chosen to ensure that only bottlenose dolphins were recorded even in the absence of visual identification^35^. Moreover, no other dolphin species are present in the area. The recordings were collected with different sampling rates (from 44 to 192 kHz). However, since the recordings collected with the lowest sampling rate (44 kHz) did not contain whistles with frequencies higher than the Nyquist frequency (22 kHz), the different sampling rate did not affect the results.

**Table 6:** Research boats and equipment used, effort and sampling periods for the six sites.

Site	Recording methods	Equipment	Effort (hours of recording)	Year
PC	2 PAM devices deployed at 25 m depth	One HTI-92-WB omnidirectional hydrophone (High Tech Inc., receiver sensitivity: − 155 dB re 1 V/μPa, flat frequency response: 2 Hz–50 kHz) and one HTI-96 omnidirectional hydrophone (High Tech Inc., receiver sensitivity: − 164 dB re 1μPa/V, flat frequency response: 2 Hz–30 kHz) connected to two EA-SDA14 solid state recorder (data format 24-bit WAV, sampling rate 78 kHz)	3 h	2014–2015
AL	Boat based survey using a 9.7 m motorboat powered by a 270 HP inboard engine. Hydrophone lowered to 5–10 m depth	Sensor Technology SQ26-08 omnidirectional hydrophone (sensitivity − 168.8 dB re 1 V/μPa; flat frequency response from 100 Hz to 30 kHz, ± 3 dB), with a bandwidth between 20 Hz and 50 kHz, connected to an M-Audio MicroTrack II, ZOOM or TASCAM recorders (data format 24-bit WAV, sampling rate 96 kHz)	27 h 49 min, over 60 days	2012–2020
FI	Boat based survey using a sailing vessel Beneteau Oceanis 41.1 powered by a 55 hp Volvo diesel engine. Hydrophones lowered to 10 m depth	Two Colmar GP0280 omnidirectional hydrophones provided by Pavia University (sensitivity − 168.8 dB re 1 V/μPa@5 kHz, flat frequency response from 1 to 30 kHz ± 5 dB) with a bandwidth between 5 Hz and 90 kHz, connected to 15 m and 40 m kevlar cables. One Audio interface Roland Quad Capture UA55. (data format 16–24-bit WAV, sampling rate 44.1, 48 and 96 kHz)	6 h 45 min	2017–2018
LA	PAM devices deployed at 35–40 m of depth	Programmable underwater acoustic recorders (M-Audio MicroTrack II) and hydrophones with bandwidth between 10 Hz and 96 kHz (Sensor Technology SQ2; sensitivity − 169 dB re 1 V/1uPa, data format 16–24-bit WAV, sampling rate 96 kHz)	119 h, over 34 days	2006–2009
GC	Boat based survey using a 5.8 m RIB powered by a four-stroke 100 HP outboard engine. Hydrophone lowered to 5 m depth	Sensor Technology SQ26-08 omnidirectional hydrophone (sensitivity − 168.8 dB re 1 V/μPa; flat frequency response from 100 Hz to 30 kHz, ± 3 dB), with a bandwidth between 20 Hz and 50 kHz connected to a Zoom H1 Digital Flash Recorder (data format 16–24-bit WAV, sampling rate 96 kHz)	3 h, over 21 days	2013–2017
CL	Boat based survey using 5.8 m RIB powered by a four-stroke 90 HP outboard engine. Hydrophone lowered to 5 m depth	RESON TC 4,032 omnidirectional hydrophone (sensitivity − 170 dB re 1 V/μPa; flat frequency response from 10 Hz to 80 kHz, ± 2.5 dB), with a bandwidth between 5 Hz and 120 kHz, connected to a SOUNDDEVICES 702 high-resolution digital audio recorder (data format 24-bit WAV, sampling rate 192 kHz; recordings down sampled at 96 kHz)	28 h 29 min, over 68 days	2016–2017

Port Cros (PC), Alghero (AL), Ostia-Fiumicino (FI), Lampedusa (LA), Gulf of Corinth (GC), Cres and Losinj (CL).

This work is based on the observation of dolphins and does not foresee any direct experiment on them. All procedures performed in the study were in accordance with the ethical standards of the involved institutions.

### Acoustic data analysis

**Figure 8:**
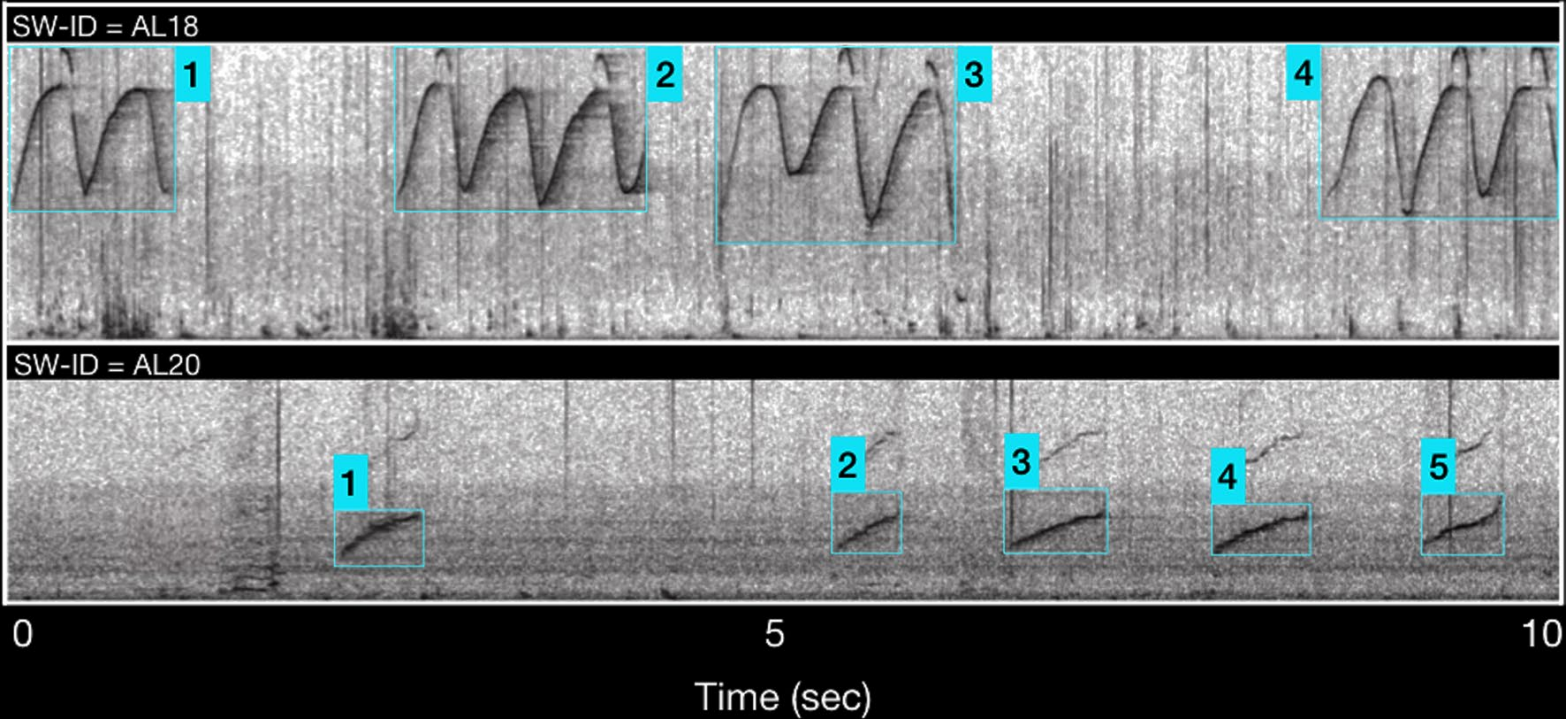
Example of SW-IDs. The number represents the stereotyped contours (referred as SW in the text) of the SW-ID.

Signature whistles were defined as “a learned, individually distinctive whistle type in a dolphin's repertoire that broadcasts the identity of the whistle owner”^14^. Thus, signature whistles of a same individual are characterized by the same frequency modulation pattern (called contour—SW). The SWs can be produced in loops (repetitions of the same elements), usually separated by intervals less than 250 ms^27^, and can also have an introductory and/or final loop^14^ distinct from the central pattern. We considered any single or multiple-loop whistle, connected or disconnected, as the unit of analysis^27^. To classify a whistle as a SW, the SIGID method^14,51^ was applied, following a step-by-step procedure. First, each whistle was graded depending on the quality and the signal-to-noise ratio (SNR) as follows: (i) whistle fairly audible and with contour not clearly discernible; (ii) whistle audible and clearly visible from the beginning to the end of the contour; (iii) whistle predominant. Weak whistles, whistles overlapping with other sounds, whistles with no good definition of the contour and with no clear start and end points (graded as 1) were discarded from the sample^35,36,52^. Following Kriesell et al.^30^, the whistles present in any recording session were distinguished as repeated element whistle type (REWT—those whistles with the same contour that are present at least twice within the range of 0.25–10 s during a recording section), and other whistle (OW—variant whistles that did not respect the previous classification rule). A catalogue containing all the REWTs was constructed, assigning to each a unique identification code and a minimum of two good images of the relative contours. Then, the recordings were inspected a second time and whistles were compared with those in the catalogue. A whistle was classified as a SW if a minimum of four stereotyped contours were present in a recorded session and 75% of them occurred within 1–10 s of at least one other^51^. When a SW was identified, a distinctive individual code was assigned (SW-ID) and any whistle with the same contour was assigned to the same SW-ID (Fig. 8).

For each SW, minimum (min) frequency, maximum (max) frequency, start frequency, end frequency, frequency range, number of inflection points and duration (as defined in La Manna et al.^36^) were measured by visual inspection of the spectrogram [512/1024-point fast Fourier transform (FFT), frequency resolution of 135 Hz, Hann window, 50% overlap] under Raven 1.5 software (Cornell Laboratory of Ornithology, Ithaca, NY, USA).

#### Region, geographic site, sea bottom and population demography

Four types of potential factors influencing SW structure were considered: region, geographic site, sea bottom type and dolphin population demography.

From a genetic point of view, a differentiation exists between the bottlenose dolphin populations of the western and eastern Mediterranean regions^37^. Based on this knowledge, the whistles collected in the six sites were assigned to three regions as follows: (i) PC, AL and FI to the western Mediterranean Sea (WEST); (ii) CL and GC to the eastern Mediterranean Sea (EAST); (iii) LA to the southern Mediterranean Sea (SOUTH). ‘Region’ is therefore a factor with three levels (WEST, EAST, SOUTH) and accounts for the effect of the genetic distance on the structure of SWs. Whistles from LA were classified as SOUTH for two reasons: (i) the Strait of Sicily where Lampedusa is located is the transition zone between the island of Sicily and the African coast which separates the east and west Mediterranean Sea; (ii) there are no genetic data of the bottlenose dolphins off LA, but a previous study found that this population is acoustically closer to the Atlantic populations, compared to the western Mediterranean populations^35^.

The six populations studied live hundreds of kilometres apart and are assumed to be isolated from each other. ‘Site’ is a factor with six levels (PC, AL, FI, LA, GC, CL) that consider the effect of geographical isolation on the structure of SWs.

Different sea bottom type (substrate, habitat, and depth) can affect the acoustic environment and the sound propagation^38^, thus they can also influence SWs. Data on depth range and preferential habitat types of the studied populations were extracted by EMODnet platform (European Marine Observation and Data Network; https://emodnet.ec.europa.eu/en). The prevalent habitat types were classified based on the EUNIS 2021 classification system (Table 5). To evaluate the similarity between the six sites based on the sea bottom type, a hierarchical cluster analysis (using the ward linkage method, Euclidian distance) was performed. Thus, ’sea bottom’ is a factor with three levels (H1, H2 and H3—see “Results” section) that accounts for the influence of depth range and habitat on SW structure.

At the end, we extrapolated six demographic variables from existing literature: mean and range of the population size, mean and range of the group size, residency pattern (prevalence of resident or transient individuals) and connection with adjacent populations (Table 5). A hierarchical cluster analysis (using the complete linkage method, Euclidian distance) was performed to evaluate the demographic similarity between the six populations. Thus, population demography is a factor with four levels (P1, P2, P3 and P4—see “Results” section) that accounts for the influence of population characteristics on SW structure.

### Statistical analysis

First, the similarity in SW repertoire among regions, sites, sea bottom and population demography types were estimated. Thus, a non-metric multidimensional scaling ordination (nMDS) was produced from the sample similarity matrix. The mean values of the acoustic characteristics of each SW were used and data were fourth root transformed before calculating the Bray–Curtis similarity. Then, a one-way non-parametric similarity analysis (Anosim) was applied on the same matrix to test the null hypothesis that there was no difference in SWs between the levels of each factor (region, site, sea bottom and population demography). To perform this analysis, the functions metaMDS and anosim of the R package Vegan^53^ were used.

When mean values of SWs are used as units of analysis, in order to respect the independence between samples, the magnitude of variability decreases. Thus, to investigate the association between the SW structure and each factor, all the SWs collected were used, and Generalized Linear Mixed Models (GLMMs) with Gaussian distribution were applied. The GLMMs are an extension of Generalized Linear Models that allow for the inclusion of random effects, by modelling the covariance structure that is generated by the grouping of data^54^. They are used when the data are not independent, such as when a variable is measured more than once from the same individuals^55^ as the SWs.

Since the seven whistle characteristics were highly collinear, before running the GLMMs, a principal component analysis (PCA) was used to reduce them into three independent variables. The assumptions (linear relation between variables, sampling adequacy, and absence of outliers) were verified and some outliers were removed from the sample. The first three components (PC1, PC2, PC3) explained 84% of the total variance (see the “Results” section) and were retained because eigenvalues for the remaining four components were all < 1 (Kaiser's criterion). To perform PCA, the function prcomp of the R package Rstats^53^ was used. Thus, with the aim to investigate the association between PC1, PC2, and PC3 (the response variables) and each factor separately, four groups of models were built using region, site, sea bottom and population demography as fixed terms, while SW-ID was considered as a random factor in the models. The best model was validated by means of graphical inspection of residuals (i.e., residuals vs. fitted values plots to verify homogeneity; Q–Q plots of the residuals for normality; and plots of residuals vs. each explanatory variable to check for independence). To perform the GLMMs, the function lme of the R package nlme^56^ was used.

## Supplementary Information

Supplementary Information 1.

Supplementary Information 2.

## Data Availability

The datasets generated during and/or analysed during the current study are available from the corresponding author on reasonable request.

## References

[1] Wilkins MR, Seddon NR, Safran RJ (2013). Evolutionary divergence in acoustic signals: causes and consequences. Trends Ecol. Evol..

[2] Wei C, Rosenfeld CS, Hoffmann F (2021). Sound production and propagation in cetacean. Neuroendocrine Regulation of Animal Vocalization.

[3] Nakakara F (2002). Social functions of cetacean acoustic communication. Fish. Sci..

[4] Caldwell MC, Caldwell DK (1968). Vocalization of naive captive dolphins in small groups. Science.

[5] Caldwell MC, Caldwell DK, Tyack PL, Leatherwood S, Reeves RR (1990). Review of the signature-whistle-hypothesis for the Atlantic bottlenose dolphin. The bottlenose dolphin.

[6] Ford JB (1991). Vocal traditions among resident killer whales (*Orcinus orca*) in coastal waters of British Columbia. Can. J. Zool..

[7] Weilgart L, Whitehead H (1997). Group-specific dialects and geographical variation in coda repertoire in South Pacific sperm whales. Behav. Ecol. Sociobiol..

[8] Deeck VB, Ford JKB, Spong P (2000). Dialect change in resident killer whales: implications for vocal learning and cultural transmission. Anim. Behav..

[9] Chen Z, Wiens JJ (2020). The origins of acoustic communication in vertebrates. Nat. Commun..

[10] Morton ES (1975). Sources of selection on avian sounds. Am. Nat..

[11] Irwin DE, Thimgan MP, Irwin JH (2008). Call divergence is correlated with geographic and genetic distance in greenish warblers (*Phylloscopus trochiloides*): A strong role for stochasticity in signal evolution?. J. Evol. Biol..

[12] Campbell P, Pasch B, Pino JL, Crino OL, Phillips M, Phelps SM (2010). Geographic variation in the songs of Neotropical singing mice: Testing the relative importance of drift and local adaptation. Evol..

[13] Connor RC, Wells RS, Mann J, Read AJ, Mann J, Connor RC, Tyack PL, Whitehead H (2000). The bottlenose dolphin: Social relationships in a fission-fusion society. Cetacean societies: Field studies of dolphins and whales.

[14] Janik VM, Sayigh LS (2013). Communication in bottlenose dolphins: 50 years of signature whistle research. J. Comp. Physiol. A.

[15] MacFarlane N, Janik V, Jensen FH, McHugh K, Sayigh L, Wells R, Tyack PL (2017). Signature whistles facilitate reunions and/or advertise identity in Bottlenose Dolphins. JASA.

[16] Buckstaff KC (2004). Effects of watercraft noise on the acoustic behaviour of bottlenose dolphins, *Tursiops truncatus*, in Sarasota Bay, Florida. Mar. Mam. Sci..

[17] Cook MLH, Sayigh LS, Blum JE, Wells RS (2004). Signature-whistle production in undisturbed free-ranging bottlenose dolphins (*Tursiops truncatus*). Proc. R. Soc. Lond. B..

[18] Watwood SL, Owen ECG, Tyack PL, Wells RS (2005). Signature whistle use by temporarily restrained and free-swimming bottlenose dolphins, *Tursiops truncatus*. Anim. Behav..

[19] Sayigh LS, Tyack PL, Wells RS, Scott MD, Irvine AB (1995). Sex difference in signature whistle production of free-ranging bottle-nosed dolphins, *Tursiops-truncatus*. Beh. Ecol. Soc..

[20] Tyack PL, Sayigh LS, Snowdon CT, Hausberger M (1997). Vocal learning in cetaceans. Social influences on vocal development.

[21] Miksis JL, Tyack P, Buck JR (2002). Captive dolphins, *Tursiops truncatus*, develop signature whistles that match acoustic features of human-made model sounds. JASA.

[22] Fripp D, Owen C, Quintana-Rizzo E, Shapiro A, Buckstaff K, Jankowski K, Wells R, Tyack P (2005). Bottlenose dolphin (*Tursiops truncatus*) calves appear to model their signature whistles on the signature whistles of community members. Anim. Cogn..

[23] Janik VM, Slater PJB (1998). Context-specific use suggests that bottlenose dolphin signature whistles are cohesion calls. Anim. Behav..

[24] Sayigh LS, Tyack PL, Wells RS, Scott MD (1990). Signature whistles of free-ranging bottlenose dolphins, *Tursiops truncatus*: mother offspring comparisons. Behav. Ecol. Sociobiol..

[25] Watwood SL, Tyack PL, Wells RS (2004). Whistle sharing in paired male bottlenose dolphins, *Tursiops truncatus*. Behav. Ecol. Sociobiol..

[26] Janik VM, Dehnhardt G, Todt D (1994). Signature whistle variations in a bottlenosed dolphin, *Tursiops truncatus*. Behav. Ecol. Sociobiol..

[27] Esch HC, Sayigh LS, Wells RS (2009). Quantifying parameters of bottlenose dolphin signature whistles. Mar. Mam. Sci..

[28] Gridley, T. Geographic and species variation in bottlenose dolphin (*Tursiops* spp.) signature whistle types. PhD Thesis Biology. University of St Andrews (2011).

[29] King SL, Janik VM (2013). Bottlenose dolphins can use learned vocal labels to address each other. Proc Natl Acad Sci USA.

[30] Kriesell H, Elwen SH, Nastasi A, Gridley T (2014). Identification and characteristics of signature whistles in wild bottlenose dolphins (*Tursiops truncatus*) from Namibia. PLoS ONE.

[31] Luis AR, Couchinho MN, dos Santos ME (2015). Signature whistles in wild bottlenose dolphins: Long term stability and emission rates. Acta Ethol..

[32] Wang DW, Würsig B, Evans WE (1995). Whistles of bottlenose dolphins: Comparisons among populations. Aquatic Mam..

[33] May-Collado LJ, Wartzok D (2008). A comparison of bottlenose dolphin whistles in the Atlantic Ocean: Factors promoting whistle variation. J. Mammal..

[34] Papale E, Azzolin M, Cascão I, Gannier A, Lammers MO, Martin VM, Giacoma C (2014). Acoustic divergence between bottlenose dolphin whistles from the Central-Eastern North Atlantic and Mediterranean Sea. Acta Ethol..

[35] La Manna G, Rako-Gospić N, Manghi M, Picciulin M, Sarà G (2017). Assessing geographical variation on whistle acoustic structure of three Mediterranean populations of common bottlenose dolphin (*Tursiops truncatus*). Beh..

[36] La Manna G, Rako-Gospić N, Sarà G, Gatti F, Bonizzoni S, Ceccherelli G (2020). Whistle variation in Mediterranean common bottlenose dolphin: The role of geographical, anthropogenic, social, and behavioral factors. Ecol. Evol..

[37] Natoli A, Birkun A, Aguilar A, Lopez A, Rus Hoelzel A (2005). Habitat structure and the dispersal of male and female bottlenose dolphins (*Tursiops truncatus*) based on microsatellite and mitochon-drial DNA analyses. Proc. R. Soc. Lond. B..

[38] Richardson WJ, Greene CR, Malme CI, Thomson DH (1995). Marine mammals and noise.

[39] Gnone, G., *et al.* TursioMed: An international project to assess the conservation status of the bottlenose dolphin in the Mediterranean Sea. Final Report (2019).

[40] La Manna, G. & Ronchetti, F. Relazione sul monitoraggio della presenza e distribuzione del tursiope *Tursiops truncatus* nell’area del nord Sardegna comprendente l’Area Marina Protetta Capo Caccia - Isola Piana. Report AMP, 42 (2018).

[41] La Manna G, Ronchetti F, Sarà G, Ruiu A, Ceccherelli G (2020). Common bottlenose dolphin protection and sustainable boating: species distribution modeling for effective coastal planning. Front. Mar. Sci..

[42] Pace DS, Giacomini G, Campana I, Paraboschi M, Pellegrino G, Silvestri M, Alessi J, Angeletti D, Cafaro V, Pavan G, Ardizzone G, Arcangeli A (2019). An integrated approach for cetacean knowledge and conservation in the central Mediterranean Sea using research and social media data sources. Aquat. Conserv..

[43] Pace DS, Di Marco C, Giacomini G, Ferri S, Silvestri M, Papale E, Casoli E, Ventura D, Mingione M, Alaimo Di Loro P, Jona Lasinio G, Ardizzone G (2021). Capitoline Dolphins: Residency patterns and abundance estimate of *Tursiops truncatus* at the Tiber River Estuary (Mediterranean Sea). Biology.

[44] Pulcini M, Pace DS, La Manna G, Triossi F, Fortuna CM (2013). Distribution and abundance estimates of bottlenose dolphins (*Tursiops truncatus*) around Lampedusa Island (Sicily Channel, Italy). Implications for their management. J. Mar. Biol. Assoc. UK.

[45] La Manna G, Ronchetti F, Sarà G (2016). Predicting common bottlenose dolphin habitat preference to dynamically adapt management measures from a Marine Spatial Planning perspective. Ocean Coast. Manag..

[46] Santostasi NL, Bonizzoni S, Bearzi G, Eddy L, Gimenez O (2016). A robust design capture-recapture analysis of abundance, survival and temporary emigration of three odontocete species in the Gulf of Corinth, Greece. PLoS ONE.

[47] Bearzi G, Bonizzoni S, Gonzalvo J (2011). Mid-distance movements of common bottlenose dolphins in the coastal waters of Greece. J. Ethol.

[48] Bearzi G, Bonizzoni S, Santostasi NL, Furey NB, Eddy L, Valavanis VD, Gimenez O, NotarbartolodiSciara G, Podestà M, Curry BE (2016). Dolphins in a scaled-down Mediterranean: The Gulf of Corinth's odontocetes. Adv. Mar. Biol..

[49] Pleslić G, Rako-Gospić N, Mackelworth P, Wiemann A, Holcer D, Fortuna CM (2015). The abundance of common bottlenose dolphins (*Tursiops truncatus*) in the former special marine reserve of the Cres-Lošinj Archipelago, Croatia. Aquat. Conserv..

[50] Rako-Gospić N, Radulović M, Vučur T, Pleslić G, Holcer D, Mackelworth P (2017). Factor associated variations in the home range of a resident Adriatic common bottlenose dolphin population. Mar. Pol. Bul..

[51] Janik VM, King SL, Sayigh LS, Wells RS (2013). Identifying signature whistles from recordings of groups of unrestrained bottlenose dolphins (*Tursiops truncatus*). Mar Mam. Sci.

[52] La Manna G, Manghi M, Pavan G, Lo Mascolo F, Sarà G (2013). Behavioural strategy of common bottlenose dolphins (*Tursiops truncatus*) in response to different kinds of boats in the waters of Lampedusa Island (Italy). Aquat. Conserv..

[53] R Core Team (2015). R: A Language and Environment for Statistical Computing.

[54] Zuur AF, Ieno EN, Walker NJ, Saveliev AA, Smith GH (2009). Mixed effects models and extensions in ecology with R, 579.

[55] Garamszegi LZ (2015). A simple statistical guide for the analysis of behaviour when data are constrained due to practical or ethical reasons. Anim. Beh..

[56] Pinheiro, J., Bates, D., DebRoy, S., Sarkar, D., & R Core Team. *nlme: Linear and Nonlinear Mixed Effects Models.* R package version 3.1–137 (2018).

[57] Janik VM (2000). Source levels and the estimated active space of bottlenose dolphin (*Tursiops truncatus*) whistles in the Moray Firth, Scotland. J. Comp. Physiol. A Sens. Neural Behav. Physiol.

[58] Quintana-Rizzo E, Mann DA, Wells RS (2006). Estimated communication range of social sounds used by bottlenose dolphins (*Tursiops truncatus*). JASA.

[59] Sayigh, L. S. Development and function of signature whistles of free ranging bottlenose dolphins, *Tursiops truncatus*. MIT/WHOI joint program (1992).

[60] Janik VM, Sayigh LS, Wells RS (2006). Signature whistle shape conveys identity information to bottlenose dolphins. PNAS.

[61] Papale E, Gamba M, Perez-Gil M, Martin VM, Giacoma C (2015). Dolphins adjust species-specific frequency parameters to compensate for increasing background noise. PLoS ONE.

[62] La Manna G, Rako-Gospić N, Manghi M, Ceccherelli G (2019). Influence of environmental, social and behavioural variables on the whistling of the common bottlenose dolphin (*Tursiops truncatus)*. Behav. Ecol. Sociobiol..

[63] Ballard SM, Lee KM (2017). The acoustics of marine sediments. JASA.

[64] Smolker R, Pepper JW (1999). Whistle convergence among allied male bottlenose dolphins (Delphinidae, Tursiops sp). Ethology.

[65] Sayigh LS, Esch HC, Wells RS, Janik VM (2007). Facts about signature whistles of bottlenose dolphins (*Tursiops truncatus*). Anim. Behav..

[66] Jourdan J., *et al.* Distribution and abundance of bottlenose dolphin (*Tursiops truncatus*) along French Provençal coast. In *Proceeding of the 30th European Cetacean Society Conference, Madeira* (2016).

[67] Labach H (2021). Distribution and abundance of common bottlenose dolphin (*Tursiops truncatus*) over the French Mediterranean continental shelf. Mar. Mam. Sci..

[68] Terranova F, Gnone G, Friard O, Bellingeri M, Giacoma C, Favaro L (2021). Signature whistles of the demographic unit of bottlenose dolphins (*Tursiops truncatus*) inhabiting the Eastern Ligurian Sea: characterisation and comparison with the literature. Eur. Zool. J..

