# Supplementary material for: Determinants of variability in signature whistles of the Mediterranean common bottlenose dolphin
Source: Sci Rep. 2022 May 26;12:6980. doi: 10.1038/s41598-022-10920-7 (PMC9135725; doi:10.1038/s41598-022-10920-7)
Supplement: Supplementary file 1 — Supplementary Information 1. [file 41598_2022_10920_MOESM1_ESM.pdf]

Supplementary Materials Table S1: number of SW contours for each SW-ID and total, per site. Port Cros (PC), Ostia-Fiumicino (FI), Alghero (AL), Lampedusa (LA), Gulf of Corinth (GC), Cres and Losinj (CL).

**PC**	**SW-IDs**	**PC1**	**PC10**	**PC11**	**PC2**	**PC3**	**PC4**	**PC5**	**PC6**	**PC7**	**PC8**	**PC9**							**Total:<br>11 SW-IDs<br>101 SW contours**
	**N° of contours**	5	10	4	4	4	7	9	30	7	18	4							
**FI**	**SW-IDs**	**FI1**	**FI10**	**FI11**	**FI12**	**FI13**	**FI14**	**FI15**	**FI16**	**FI17**	**FI2**	**FI3**	**FI4**	**FI5**	**FI6**	**FI7**	**FI8**	**FI9**	**Total:<br>17 SW-IDs<br>166 SW contours**
	**N° of contours**	9	6	6	5	8	6	9	4	6	14	17	10	6	30	11	14	5	
**AL**	**SW-IDs**	**AL1**	**AL10**	**AL11**	**AL12**	**AL13**	**AL14**	**AL15**	**AL16**	**AL17**	**AL18**	**AL19**	**AL20**	**AL21**	**AL22**	**AL23**	**AL24**	**AL25**	**Total:<br>58 SW-IDs<br>925 SW contours**
	**N° of contours**	29	31	5	46	5	36	8	11	24	83	4	12	6	17	10	16	4	
	**SW-IDs**	**AL26**	**AL27**	**AL28**	**AL29**	**AL3**	**AL30**	**AL31**	**AL32**	**AL33**	**AL34**	**AL35**	**AL36**	**AL37**	**AL38**	**AL39**	**AL4**	**AL40**	
	**N° of contours**	52	75	4	16	7	7	4	5	29	12	8	27	6	4	4	13	25	
	**SW-IDs**	**AL41**	**AL43**	**AL44**	**AL45**	**AL46**	**AL47**	**AL48**	**AL49**	**AL5**	**AL50**	**AL51**	**AL52**	**AL53**	**AL54**	**AL55**	**AL56**	**AL57**	
	**N° of contours**	11	44	8	4	5	4	6	4	9	33	7	6	6	23	13	4	20	
	**SW-IDs**	**AL58**	**AL2**	**AL6**	**AL7**	**AL8**	**AL9**												
	**N° of contours**	4	4	7	21	4	33												
**LA**	**SW-IDs**	**LA1**	**LA10**	**LA11**	**LA12**	**LA13**	**LA14**	**LA15**	**LA16**	**LA17**	**LA18**	**LA19**	**LA2**	**LA20**	**LA21**	**LA22**	**LA23**	**LA24**	**Total:<br>37 SW-IDs<br>406 SW contours**
	**N° of contours**	9	9	15	25	8	17	21	4	6	8	11	6	8	11	5	7	18	
	**SW-IDs**	**LA25**	**LA26**	**LA27**	**LA28**	**LA29**	**LA3**	**LA30**	**LA31**	**LA32**	**LA33**	**LA34**	**LA35**	**LA36**	**LA37**	**LA4**	**LA5**	**LA6**	
	**N° of contours**	5	6	4	6	4	4	8	47	5	8	6	4	4	4	13	34	12	
	**SW-IDs**	**LA7**	**LA8**	**LA9**															
	**N° of contours**	31	9	4															
**GC**	**SW-IDs**	**GC1**	**GC10**	**GC11**	**GC12**	**GC13**	**GC2**	**GC3**	**GC5**	**GC6**	**GC7**	**GC8**	**GC9**						**Total:<br>12 SW-IDs<br>83 SW contours**
	**N° of contours**	7	11	4	4	4	9	17	8	7	4	4	4						
**CL**	**SW-IDs**	**CL1**	**CL10**	**CL11**	**CL12**	**CL13**	**CL14**	**CL15**	**CL16**	**CL17**	**CL18**	**CL19**	**CL2**	**CL20**	**CL21**	**CL22**	**CL23**	**CL24**	**Total:<br>33 SW-IDs<br>346 SW contours**
	**N° of contours**	7	4	6	7	14	4	4	24	11	4	7	14	14	5	4	37	20	
	**SW-IDs**	**CL25**	**CL26**	**CL27**	**CL28**	**CL29**	**CL3**	**CL30**	**CL31**	**CL32**	**CL33**	**CL4**	**CL5**	**CL6**	**CL7**	**CL8**	**CL9**		
	**N° of contours**	18	7	12	4	6	23	4	20	4	9	6	10	8	15	4	10		
