# Supplementary material for: Determinants of variability in signature whistles of the Mediterranean common bottlenose dolphin
Source: Sci Rep. 2022 May 26;12:6980. doi: 10.1038/s41598-022-10920-7 (PMC9135725; doi:10.1038/s41598-022-10920-7)

Supplementary Materials Fig. S1: Examples of SWs from the catalogue of: a) PC; b) AL; c) FI; d) LA; e) GC; f) CL.

a)

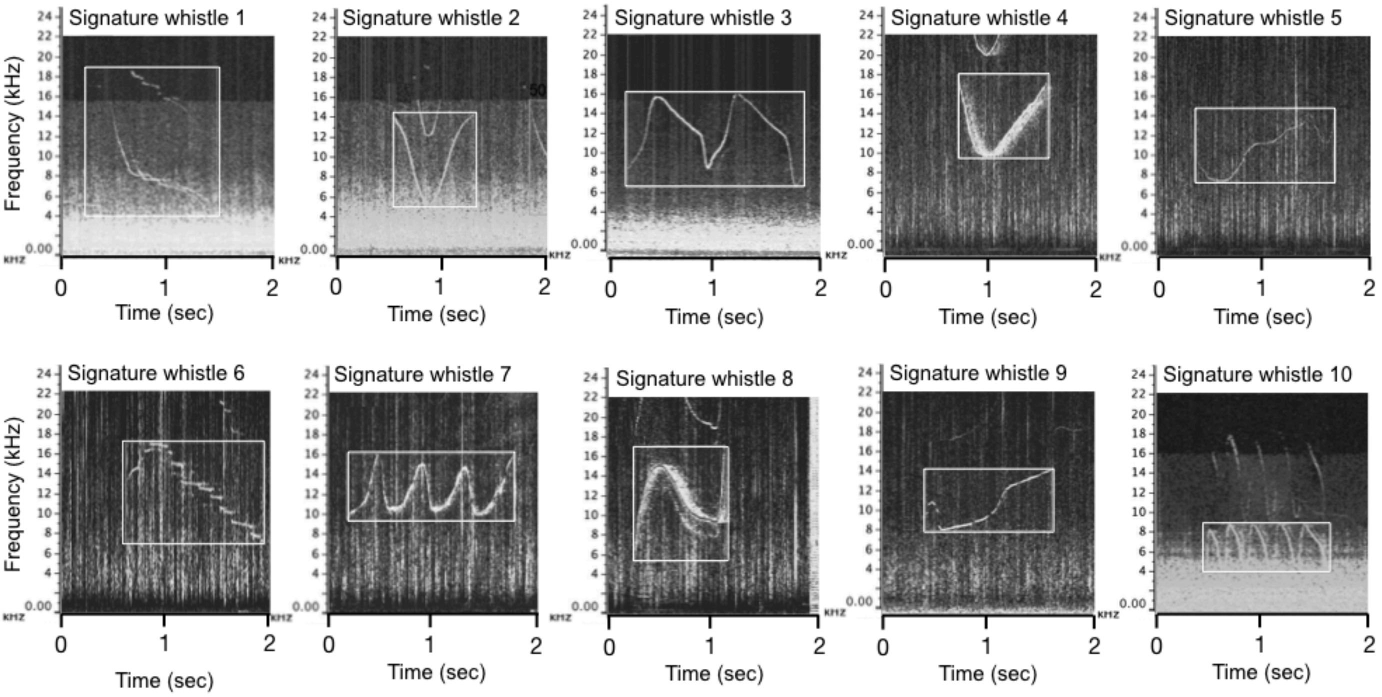

b)

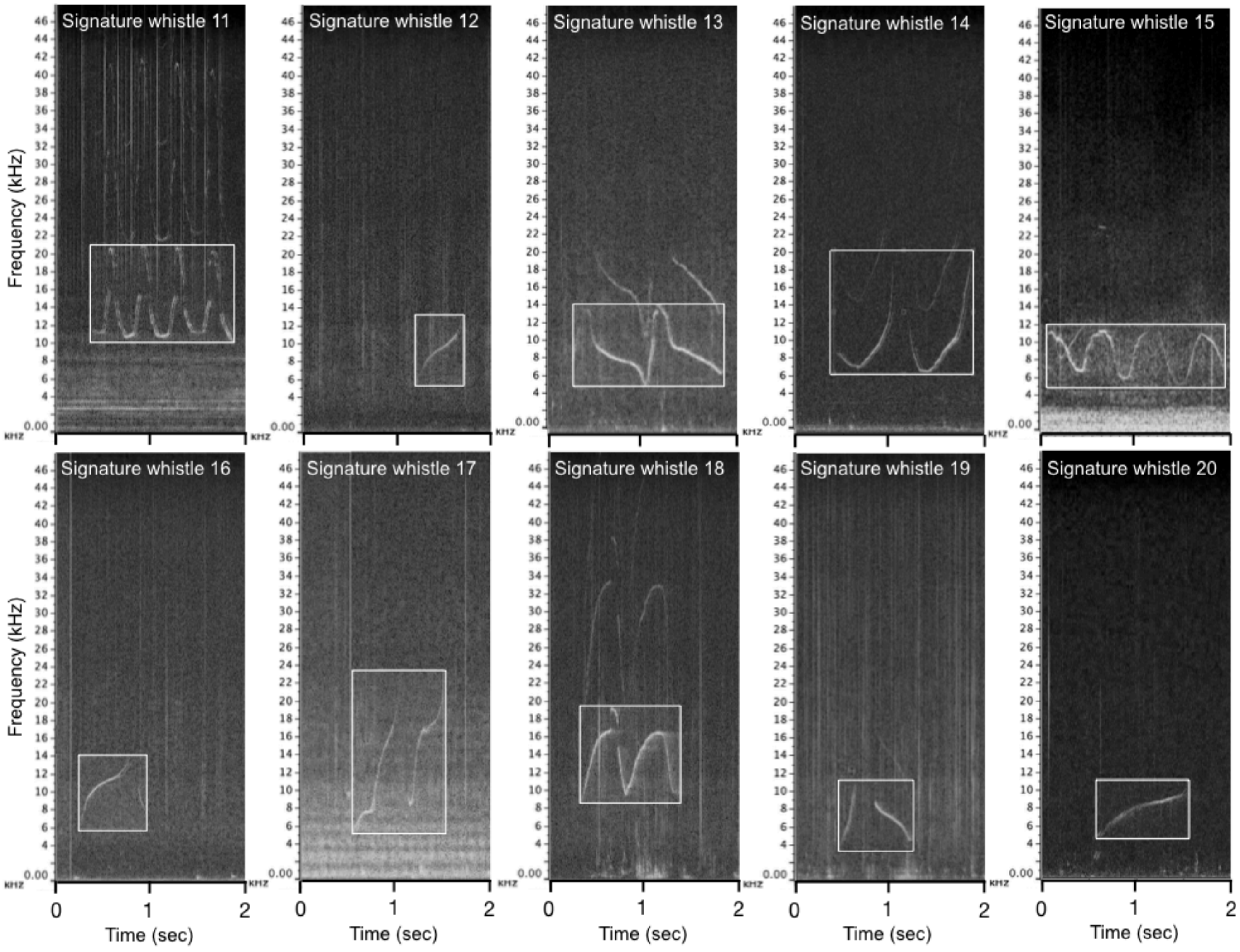

Supplementary Materials Fig. S1: Examples of SWs from the catalogue of: a) PC; b) AL; c) FI; d) LA; e) GC; f) CL.

c)

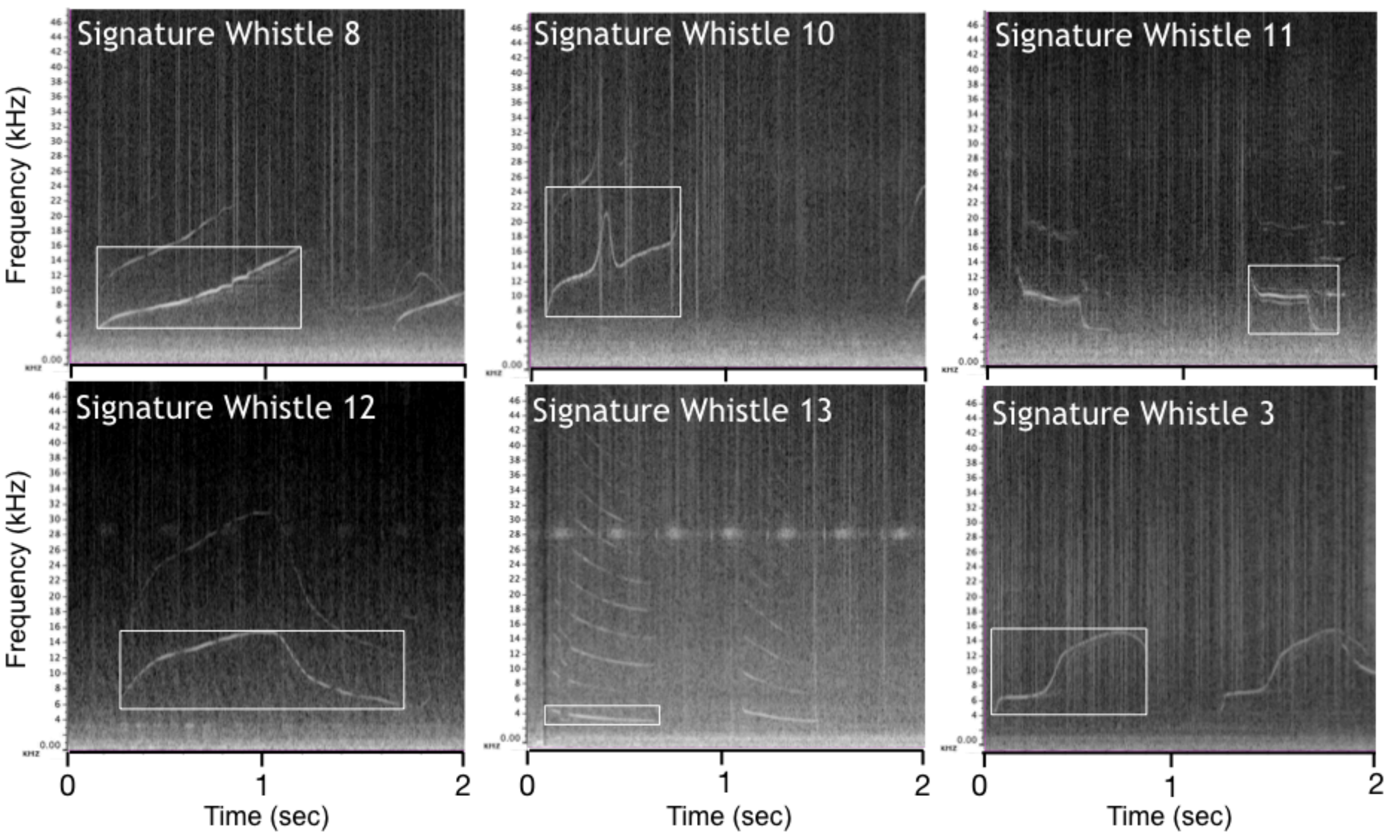

d)

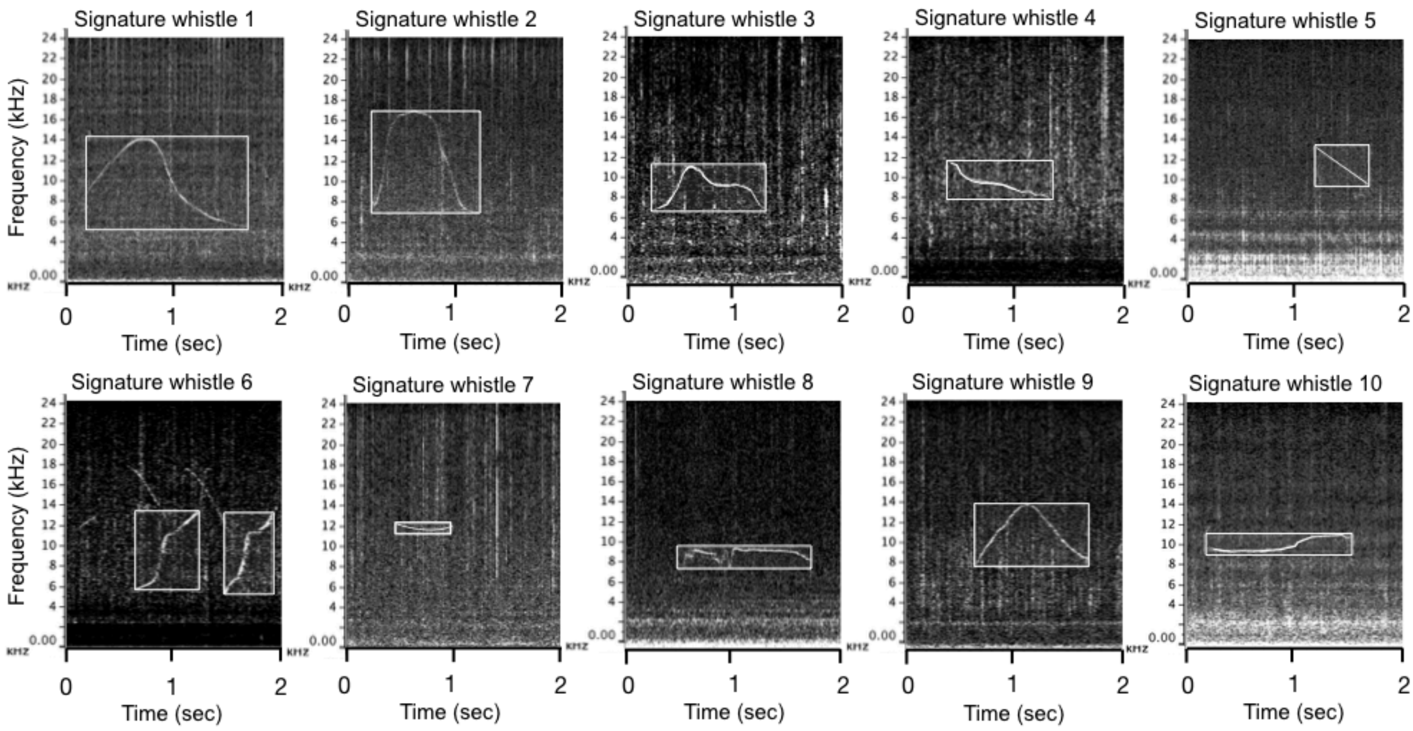

Supplementary Materials Fig. S1: Examples of SWs from the catalogue of: a) PC; b) AL; c) FI; d) LA; e) GC; f) CL.

e)

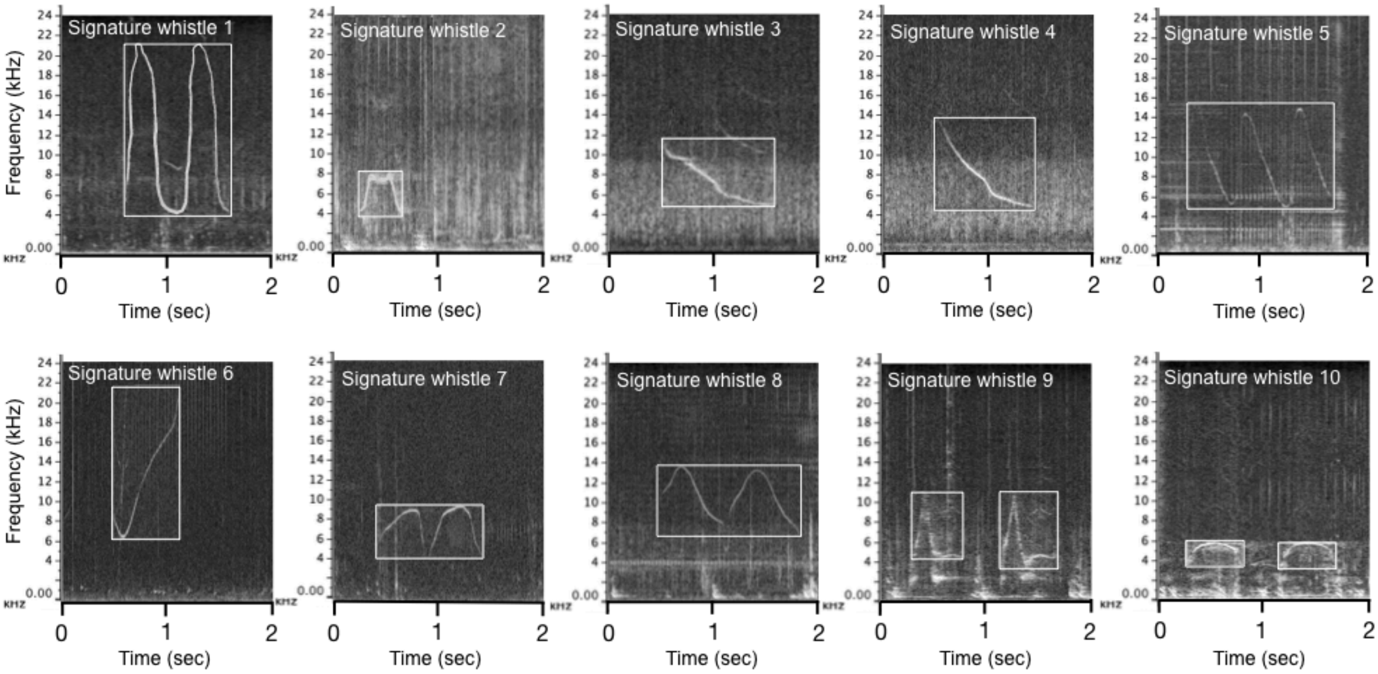

f)

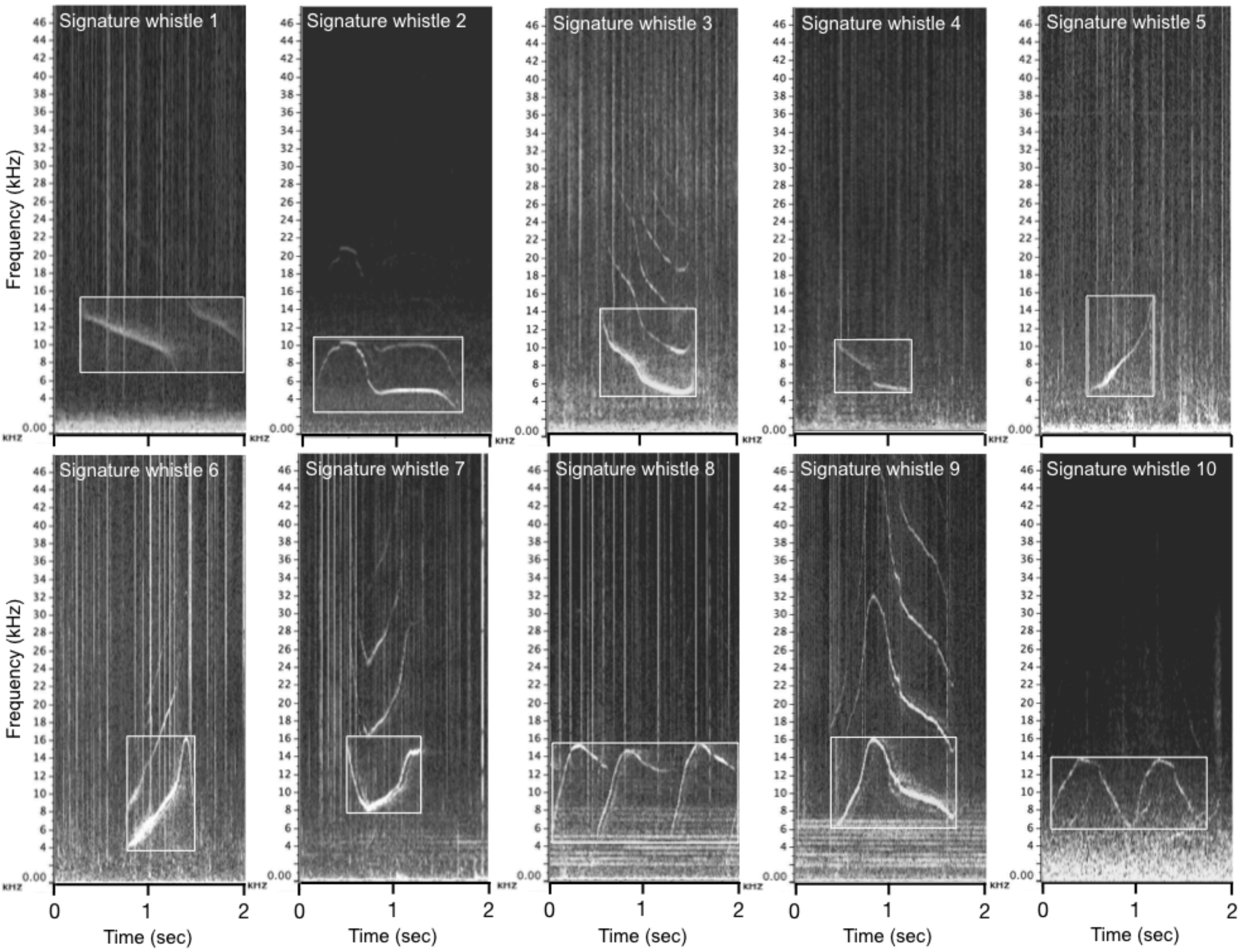

Supplement: Supplementary file 2 — Supplementary Information 2. [file 41598_2022_10920_MOESM2_ESM.pdf]
